# Antipsychotic effects of aqueous lyophilisate of *Ficus mucuso* on ketamine-induced schizophrenia

**DOI:** 10.3389/fneur.2026.1788148

**Published:** 2026-04-07

**Authors:** Fleur Clarisse Okomolo Moto, Justin Diapa Tchiengang, Jacqueline Stéphanie Kameni Njapdounke, Alain Mbom, Franklin Mbeboh Savo, Mirlene Ndawakai Aren Arsa’a, Frédérique Nadia Nezoumne, Sebastien Roger Ndjock, Elisabeth Ngo Bum

**Affiliations:** 1 Department of Biological Sciences, Higher Teacher Training College of Yaoundé, University of Yaoundé I, Yaoundé, Cameroon; 2 Department of Biological Sciences, Faculty of Sciences, University of Maroua, Maroua, Cameroon; 3 Department of Biological Sciences, Faculty of Sciences, University of Ngaoundéré, Ngaoundéré, Cameroon; 4 Department of Animal Biology and Conservation, Faculty of Sciences, University of Buea, Buea, Cameroon

**Keywords:** *Ficus mucuso*, ketamine, neurochemistry alteration, neurodegeneration, oxidative stress, risperidone, schizophrenia

## Abstract

Nutraceuticals and medicinal plants have been studied for the treatment of schizophrenia. *Ficus mucuso* is a medicinal plant used in Cameroon to treat epilepsy, jaundice, and schizoaffective disorders. Schizophrenia is a heterogeneous and ubiquitous neuropsychiatric disorder associated with neurochemical and oxidative disturbances characterized by the appearance of a triad of psychotic symptoms. This study investigated the antipsychotic effect of the aqueous lyophilisate of *F. mucuso* on behavioral disturbances, oxidative and neurochemical imbalances, and ketamine-induced neurodegeneration in white mice. In the curative approach of the study, mice received a single daily dose of ketamine (20 mg/kg, i.p.) for 14 days and were then treated 30 min later with the aqueous lyophilisate of *F. mucuso* (25, 50 and 100 mg/kg, p.o.) or risperidone from days 8 to 14. Behavioral deficits were measured using stereotyped climbing, open field, forced swimming, and Y-maze tests, followed by sacrifice. Neurochemical and oxidative imbalances were assessed by spectrophotometry in the hippocampus, prefrontal cortex, and striatum. Histological sections of brain structures were analyzed and cell counts were performed. *F. mucuso* improved behavioral abnormalities and memory deficits in ketamine-treated mice. In addition, it reversed ketamine-induced oxidative stress by significantly increasing (*p* < 0.001) glutathione, catalase, and superoxide dismutase activity and significantly decreasing (*p* < 0.001) malondialdehyde and nitrite oxide levels in the hippocampus, prefrontal cortex and striatum. Similarly, it significantly reduced (*p* < 0.001) the concentration of dopamine, serotonin, acetylcholinesterase activity, and GABA-T, and significantly increased (*p* < 0.001) the concentration of GABA and glutamate in the hippocampus, prefrontal cortex, and striatum. Histology showed that *F. mucuso* protected cellular structures by reducing neurodegeneration and increasing the number of nucleated cells. In conclusion, *F. mucuso* improved ketamine-induced neurobehavioral deficits and neurodegeneration by modulating neurotransmitters, increasing the antioxidant system, and restoring the integrity of cellular structures.

## 1 Introduction

Schizophrenia is a neuropsychiatric disorder with multiple causes involving multiple pathologies. It affects approximately 1% of the global population, with a higher prevalence among male adolescents (1, 2). Schizophrenia manifests itself through positive symptoms (hallucinations and delusions), negative symptoms (depressive mood, lethargy, and social withdrawal), and cognitive disorders (learning and memory disorders) (1, 3–5). The course of the disease is often prolonged, accounting for more than half of all psychiatric hospitalizations and placing a heavy burden on society and families. Despite the increase in the number of cases of schizophrenia, its pathophysiology remains poorly understood. However, the pathophysiological hypothesis of schizophrenia is linked to neuroinflammation, neurochemical imbalances, and oxidative stress, which alter several biological processes and pathways in the hippocampus, prefrontal cortex, and striatum, thereby affecting the structure, growth and function of neurons (4, 6). For decades, the etiology of schizophrenia has been associated with dysfunction of neurotransmitter systems, particularly the hyperdopaminergic and hypodopaminergic systems of the mesolimbic and mesocortical systems and the glutamatergic system (7–10). Taken together, these remain the main mechanisms explaining the pathophysiology and certain behavioral correlations observed during the course of the disease. However, changes in GABAergic, cholinergic, noradrenergic, and serotonergic neurotransmission have also been identified as promoters of psychotic symptoms (11, 12). In addition, there is also altered cortical dopaminergic and GABAergic neurotransmission as well as neurotrophic changes, mainly due to glutamatergic excitotoxicity and neuroinflammation (4, 13). Ketamine-induced schizophrenic phenotypes, characterized by neuroimmune and neurochemical deficiencies, have led to advances in understanding the disease (14, 15). Studies have revealed that administration of ketamine, an N-methyl-D-aspartate (NMDA) antagonist, induced NMDA receptor hypofunctionality (7, 16). In addition, results from post mortem brain imaging studies have shown variations in brain levels of dopamine, glutamate, GABA, serotonin, and acetylcholine (17). These results also indicate a change in the expression of synaptic dopamine receptor proteins (D1 and D2), a decreased in serotonergic receptors (5-HT1A and 5-HT2A), metabotropic GABA-B, and muscarinic receptors (18). There is evidence of ketamine-induced alteration of the nicotinic 7-alpha acetylcholine receptor (*α* − 7nACh) and a reduction in cholinergic transmission caused by NMDA receptor antagonism. This further confirms the involvement of cholinergic transmission disturbances in psychotic disorders such as schizophrenia (11, 12, 18, 19). Specific mechanisms of ketamine have been associated with impaired GABAergic inhibitory control of NMDA receptors and alterations in the stabilizing effects of 5-HT1A and 5-HT2A receptors on dopamine, glutamate, and serotonin release, particularly in the hippocampus, prefrontal cortex, and striatum (7, 18). Consequently, modulations of 5-HT1A and 5-HT2A receptors mediated the antipsychotic effects of second-generation drugs such as risperidone. This is explained by their ability to reduce excessive neuronal excitability through hyperpolarization of pyramidal neurons in the prefrontal cortex (5). These brain regions have been implicated in the circuits of behavioral phenotypes during the process of neuronal maturation and the psychobehavioral disorders observed in schizophrenia (20). Thus, modulation of neurochemical signaling, associated with the oxidative stress pathway, and neurodegeneration has been considered a plausible mechanism for reversing ketamine-induced schizophrenic pathology (21, 22). In addition to the use of antipsychotic drugs to treat schizophrenia (23), plants are now considered an alternative and maintenance treatment due to their availability and low cost (24), as well as their ability to alleviate schizophrenia with few or no adverse effects. Our attention has been drawn to *F. mucuso*, a medicinal plant of the Moraceae family native to the Middle East. It is found in Asia and Africa, particularly in humid tropical areas (25, 26). It is rich in polyphenols, cardenolides, triterpenoids, steroids, saponins, alkaloids, flavonoid polyphenols, and tannins (27, 28). It has remarkable neuroprotective functions, including antioxidant (28, 29), anti-schizoaffective (30), hepatoprotective, nephroprotective (27), antimicrobial (31), anticancer (32), antidiabetic (33), and antiepileptic effects (34). Could *F. mucuso* have antipsychotic effects? In this study, we explored in more detail the neurochemical modulatory effects of *F. mucuso* on psychotic-like behaviors associated with changes in neurotransmitters, markers of oxidative stress, and neurodegeneration induced by ketamine using a curative approach in mice.

## 2 Materials and methods

### 2.1 Plant material

The plant material used in this study consists of bark from the trunk of *F. mucuso*, harvested in Cière, a town located approximately 19 km from Ngaoundéré, in the department of Vina, Adamaoua region. Botanical identification was carried out at the National Herbarium of Cameroon (HNC) in Yaoundé by comparison with the collection of A.J.M. Leeuwemberg (No. 9668), under registration number 44030/HNC.

#### 2.1.1 Extraction protocol

The bark of *F. mucuso* was cleaned of impurities, then washed with water and dried in the shade. After drying, it was ground and sieved to obtain a fine powder. A mass of 500 g of this powder was added to 5,000 mL of distilled water. The mixture was boiled at 100 °C for 20 min and then filtered through Whatman No. 1 paper. The filtrate obtained was frozen at −40 °C and freeze-dried using a rotary evaporator (CHRIST, ALPHA 2–4 LO) at 45 °C to extract the water. A dry mass of 139.40 g of crude extract was obtained at the end of the process, representing a yield of 27.88%.

### 2.2 Animal material

Sixty naive albino mice weighing between 20 and 30 g were used for this study. They were raised in the animal facility at the University of Ngaoundéré and fed a standard diet consisting of corn bran, corn flour, wheat flour, fish meal, peanut meal, soybean meal, palm oil, table salt, and drinking water.

The experiments were conducted in accordance with European Union guidelines on ethics in animal experimentation (EEC Council No 86/609), with the approval of the ethics committee of the institutions of the Cameroon Ministry of Scientific Research and Innovation.

### 2.3 Chemical materials

Various chemicals were used in this study, including:

- Ketamine hydrochloride (KET) (20 mg/kg, i.p.) – SynMex Pharma, India.
- Risperidone (RIS) (0.5 mg/kg, p.o.) – Sigma Aldrich, Germany.

## 3 Distribution and treatment of mice

Sixty mice were divided into six homogeneous groups (*n* = 10). All solutions were administered in a volume of 10 mL/kg body weight. With the exception of the normal control group, the mice received ketamine followed by *F. mucuso* lyophilisate for the test groups and risperidone for the positive control group for 14 consecutive days, starting on the 8th to 14th day, 30 min after administration of the inducer, according to the following distribution:

- Group 1 (normal control): Saline solution (NaCl, 0.9%, i.p.) for 14 days. Saline solution (NaCl, 0.9%, p.o.) from the 8th to the 14th day, 30 min after;
- Group 2 (negative control): Ket (20 mg/kg, i.p.) for 14 days. Saline solution (NaCl, 0.9%, p.o.) from the 8th to the 14th day, 30 min after;
- Group 3 (positive control): Ket (20 mg/kg, i.p.) for 14 days. Risperidone (0.5 mg/kg, p.o.) from day 8 to day 14, 30 min after;
- Group 4: Ket (20 mg/kg, i.p.) for 14 days. Aqueous lyophilisate of *F. mucuso* (25 mg/kg, p.o.) from day 8 to day 14, 30 min after;
- Group 5: Ket (20 mg/kg, i.p.) for 14 days. Aqueous lyophilisate of *F. mucuso* (50 mg/kg, p.o.) from day 8 to day 14, 30 min after;
- Group 6: Ket (20 mg/kg, i.p.) for 14 days. Aqueous lyophilisate of *F. mucuso* (100 mg/kg, p.o.), from day 8 to day 14, 30 min after.

**Figure 1:**
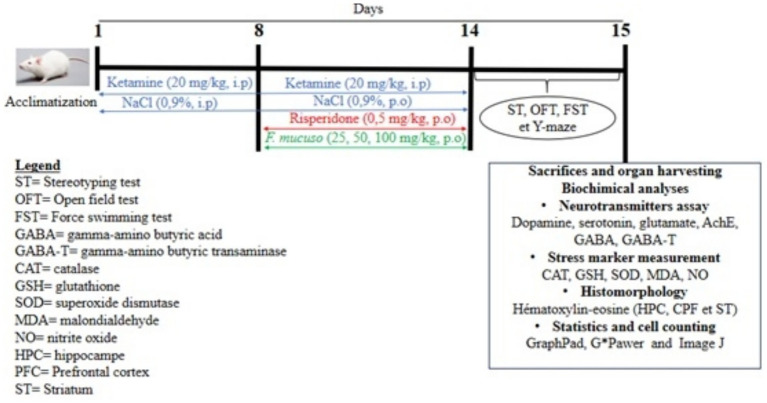
Experimental design.

Psychotic symptoms such as hyperlocomotion, despair, and memory deficits were assessed using behavioral tests from day 14 to day 15 according to the experimental design shown below (Figure 1). The number of mice per batch was chosen based on the power test adopted in accordance with the work of (35) in behavioral neuroscience. The power analysis was performed using G*Power software (version 3.1.9.7) for one-way ANOVA with six batches of 10 mice per batch (*n* = 60) followed by a *post hoc* test. With [*F*(5, 54) = 24.57], an effect size of 0.64, corresponding to a large effect according to Cohen’s criteria, and a significance threshold of *α* = 0.05 with a power of 97% (1 − *β* = 0.97) and a critical value of *F* = 2.38.

## 4 Pharmacological tests

### 4.1 Stereotypical climbing test

Stereotypy was assessed on the basis of ritualistic, repetitive and nonfunctional motor behaviors (1). The method used was that described and adopted by (36). After induction and treatment, 30 min later the mice were immediately placed in a cylindrical mesh cage or in a transparent observation chamber (40 cm × 40 cm × 19 cm). The number and duration of climbs were then recorded using a camera (1,080 pixels) for 2 min at 15, 30, 45, and 60 min (37). The observation chamber was cleaned with 70% ethanol after each test session.

### 4.2 Open arena test

The open arena test was used to observe the positive symptoms of schizophrenia in mice (10, 37, 38). The experiment was conducted in a square wooden box (40 × 40 × 19 cm), the floor of which was divided into 17 visible 10 cm^2^ squares, including a central square. The method used was that described by (14). Each mouse was placed individually in the center of the arena and left to explore freely for 5 min. Behavioral parameters, including the number of lines crossed and the duration of immobility and ambulation, were recorded by a camera (1,080 pixels). After each run, the device was cleaned with a 70% hydroethanolic solution to prevent neophobia.

### 4.3 Forced swim test

The forced swim test is an approved model for assessing despair and detecting depressive behaviors in mice (39). It is also predictive of the negative symptoms of schizophrenia. The device consisted of a cylindrical Plexiglas tank (25 cm high, 10 cm in diameter) filled with water (15 cm high) maintained at a temperature of 25 ± 2 °C. The methodology used is that described by (40). The test was conducted in two phases:

- Pre-test phase: each mouse was placed in the tank and forced to swim for 5 min.
- Test phase: 24 h later, each mouse was immersed again for a 6-min swimming session.

The behaviors captured by a camera (1,080 pixels) included immobility time (no movement for at least 2 s or movements limited to keeping the nose out of the water), swimming time, and climbing time. The test excluded the first 2 min, during which the animal generally tries to escape (10, 37, 40). A mouse exhibiting despair behavior spent more time immobile than a normal mouse.

### 4.4 Y-maze test

The Y-maze test is commonly used to assess short-term memory in rodents. This test analyzes spatial working memory as an indicator of cognitive dysfunction associated with schizophrenia, based on the percentage of alternations (18, 41, 42). The device is a wooden maze consisting of three identical arms (35 × 15 × 10 cm), symmetrically separated by an angle of 120° (43). The methodology applied is that of (37). Each mouse was placed at the end of arm A and allowed to freely explore the three arms (A, B, C). For 5 min, the number of visits to each arm and the alternations were recorded via a camera (1,080 pixels). The percentage of alternations was calculated using the following formula:


$$\%\mathrm{AS}=[\begin{array}{l} (\text{Number of alternations})/\\ \left(\text{Total number of entries into arms}-2\right) \end{array}]\times 100$$

An alternation corresponds to a consecutive entry into three distinct arms (ABC, CAB, or BCA). After each session, the device was cleaned with 70% alcohol to remove any residual odors.

## 5 Sacrifice, organ removal for histology, and homogenate preparation

### 5.1 Sacrifice and homogenate preparation

Immediately after behavioral testing, mice from each batch were decapitated. Brains were dissected on ice. The striatum, hippocampus, and prefrontal cortex were isolated and weighed. These structures were crushed, placed in tubes, and homogenized with phosphate buffer (0.1 M; pH 7.4) at 10% m/v. Each homogenized brain tissue sample was centrifuged at 10,000 rpm for 15 min (BioLAB, 3 °C). The supernatant was collected using a micropipette and stored in Eppendorf tubes at −80 °C in the refrigerator for various biochemical tests.

### 5.2 Preparation of brains for histology

The bark of *F. mucuso* was cleaned of impurities, then washed with water and dried in the shade. After drying, it was ground and sieved to obtain a fine powder. A mass of 500 g of this powder was added to 5,000 mL of distilled water. The mixture was boiled at 100 °C for 20 min and then filtered with Whatman No. 1 paper. The filtrate obtained was frozen at −40 °C and freeze-dried using a rotary evaporator (CHRIST, ALPHA 2–4 LO) at 45 °C to extract the water. A dry mass of 139.40 g of crude extract was obtained, representing a yield of 27.88%.

## 6 Evaluation of neurotransmitter concentrations

### 6.1 Dopamine assay

In the presence of hydrochloric acid, dopamine oxidizes to form an indole derivative that binds to trihydroxyindoles and forms a fluorescent complex, whose absorbance at 485 nm is proportional to the concentration of dopamine (44). A volume of 2.5 mL of heptane and 0.31 mL of HCl (0.1 M) were introduced into a dry tube. Then, 1 mL of the homogenate/dopamine (0, 300, 600, 800, 1,000, 1,200, and 1,400 μg/mL) previously prepared with HCl-butanol was added, and the mixture was vigorously shaken for 10 min using a vortex mixer. The tubes were then centrifuged at 3,000 rpm at 0 °C for 15 min to separate the two phases. The supernatant (0.2 mL) was collected and the samples were read at 485 nm using a spectrophotometer.

### 6.2 Serotonin assay

In the presence of hydrochloric acid, serotonin oxidizes to form an indole derivative. This derivative binds to O-phthaldialdehyde to form a fluorescent complex, whose absorbance at 470 nm is proportional to the concentration of serotonin. The serotonin level was estimated using the method described by (44). A volume of 0.08 mL of the homogenate was taken and placed in a tube containing 0.2 mL of heptane and 0.025 mL of hydrochloric acid (0.1 M). After vigorous shaking of the tubes for 10 min using a vortex mixer, the mixture was centrifuged at 3,000 rpm at 0 °C for 15 min to separate the two phases. The upper organic phase was then removed and the aqueous phase retained for serotonin estimation. Thus, 0.2 mL of the aqueous phase was introduced into dry tubes and 0.25 mL of O-phthaldialdehyde reagent was added, then the mixture was heated to 70 °C for 10 min. The absorbance was read at 470 nm using a spectrophotometer.

### 6.3 Glutamate assay

In the presence of NAD^+^, L-glutamate is oxidized to *α*-ketoglutarate, catalyzed by glutamate dehydrogenase (GDH). This method allows for direct, sensitive, and specific quantification of glutamate (45).

Glutamate + NAD^+^ + H₂O → α-ketoglutarate + NH₄^+^ + NADH + H^+^. In each well containing 0.8 mL of phosphate buffer solution (0.1 M, pH 7.4), 0.1 mL of NAD^+^ (nicotinamide adenine dinucleotide, oxidized form 10 mM), 0.05 mL of sample, and finally glutamate dehydrogenase enzyme (0.05 mL) were added successively. After vigorous shaking of the tubes using a vortex mixer, the mixture was heated in a water bath at 37 °C for 5 min. The absorbance was read at 340 nm using a spectrophotometer. The glutamate concentration was calculated from the variation in absorbance at 340 nm with Ɛ = 6,220 L·mol^−1^·cm^−1^.

### 6.4 Measurement of gamma-aminobutyric acid

In a basic medium, the reaction between ninhydrin and gamma-aminobutyric acid (GABA) will produce a purplish-red color. The absorbance between 377 nm and 530 nm is proportional to the concentration of GABA in the sample. The amount of GABA was evaluated using the colorimetric assay technique for mouse brain homogenates described by Lowe et al. (46). 0.2 mL of 0.14 M ninhydrin solution was prepared in a bicarbonate buffer solution (0.5 M; pH 9.9), and 0.1 mL of 10% glacial trichloroacetic acid (TCA). 100 μL of sample was added to the medium and the mixture was incubated at 60 °C in a water bath for 30 min. After cooling, the mixture was added to 5 mL of copper tartrate solution prepared from 0.16% disodium carbonate, 0.03% copper sulfate, and 0.0329% tartaric acid, maintained at a temperature of 25 °C for 10 min. The fluorescence resulting from the reaction between ninhydrin and GABA in the basic medium was measured using a spectrofluorometer. The absorbance measured was proportional to the concentration of GABA in the homogenates.

### 6.5 Measurement of gamma-aminobutyric acid transaminase

In the presence of iron chloride, succinic semialdehyde and 3-methyl-2-benzothiazol-2-hydrazone form a colored complex, whose absorbance at 610 nm is proportional to the activity of GABA transaminase. GABA-T activity was assessed using the colorimetric assay method (47). 15 μmol of α-ketoglutarate, 15 μmol of GABA, and 10 μmol of pyridoxal-5-phosphate were added to tubes, then 0.1 mL of homogenate was added to the test tubes while 0.1 mL of 5% methanol was added to the blank tubes. The final volume was made up to 3 mL with Tris–HCl buffer (50 Mm; pH 7.4) and the tubes were incubated at 37 °C for 60 min in a water bath. Finally, 0.5 mL of TCA (20%) and 0.1 mL of ferric chloride III (12%) were added to each tube, and the absorbance was read at 610 nm at 30 and 90 s against the blank. The enzymatic activity of GABA-T was determined in μg/min/mg of tissue according to Beer–Lambert’s law (Ɛ = 40 M^−1^.cm^−1^).

### 6.6 Acetylcholinesterase assay

Ellman’s reagent or DTNB (5,5′-dithiobis (2-nitrobenzoic acid)) is a chromogen in an oxidation–reduction reaction following the enzymatic reduction of acetylthiocholine to thiocholine. The increase in yellow coloration indicates the formation of thiocholine, which reduces DTNB to TNB (5-thio (2-nitrobenzoic acid)), reflecting the activity of the enzyme with a maximum absorbance at 412–415 nm (48). In 925 μL of 2,2′-dithio-5,5′-dinitrobenzoic acid (DTNB), 50 μL of acetylthiocholine iodide and 25 μL of Tris buffer were added, followed by 25 μL of homogenates. The reaction medium was thoroughly mixed by bubbling air through it. The gradual change in absorbance was measured by a spectrophotometer at 412 nm for 3 min at 30-s intervals. Enzyme activity is expressed in μmol/min/mg of protein/min (9). One unit is defined as 1 mole of acetylthiocholine hydrolyzed per minute per milligram of protein. The enzymatic activity of acetylcholinesterase is calculated according to Beer–Lambert’s law (Ɛ = 13,600 mole/cm), which corresponds to the molar extinction coefficient of the yellow compound formed (49).

## 7 Estimation of oxidative stress markers in the hippocampus, prefrontal cortex, and striatum

Pro-oxidant and antioxidant molecules were sought in the supernatant of the HPC, PFC, and ST. Pro-oxidants in the brain regions were measured by direct determination of MDA peroxidation markers and nitric oxide (NO) concentration. Antioxidant enzymes (SOD and CAT) were estimated based on the inhibition of adrenaline superoxide, and finally a non-enzymatic antioxidant (GSH) was estimated using the Ellman method.

### 7.1 Catalase assay

Hydrogen peroxide is broken down in the presence of catalase. The residue binds to potassium dichromate to form a blue-green precipitate of unstable perchloric acid, which decomposes when heated to form a green complex. Catalase activity was measured using the method described in (50). 50 μL of tissue homogenate was added to 750 μL of phosphate buffer (0.1 mM, pH 7.5). Next, 200 μL of H_2_O_2_ (50 mM) was added to the test tubes. One minute later, 2,000 mL of potassium dichromate (5%) prepared in 1% glacial acetic acid was added to the reaction medium. The tubes were then incubated at 100 °C for 10 min in a water bath and then cooled to 25 °C. The optical densities were recorded at 570 nm. The catalase level in the samples was obtained from a previously established calibration curve. The specific activity of catalase is expressed in Units/mg of protein.

### 7.2 Measurement of reduced glutathione in the brain

Dinitro-2,2′-dithio-5,5′-dibenzoic acid (DTNB) reacts with the thiol (-SH) groups of glutathione and forms a yellow-colored complex with maximum absorption at 412 nm (51). In advance, 100 μL of homogenates (sample tubes) or 100 μL of 50 mM Tris–HCl buffer, pH = 7.4 (control tube), were introduced into the test tubes, followed by 1,500 μL of Ellman’s reagent. The tubes were shaken and incubated for 60 min at room temperature, and the absorbance was read at 412 nm. The glutathione concentration was calculated using the molar extinction coefficient (*ε* = 13,600 mol^−1^.cm^−1^).

### 7.3 Superoxide dismutase assay

The oxidation of adrenaline to adrenochrome in a medium is inhibited in the presence of superoxide dismutase (SOD). The increase in absorbance is proportional to SOD activity and is recorded between 20 and 80 s at 480 nm. Tissue SOD activity was determined using the method described by Misra and Fridovich (52). 134 μL of homogenate was added to the test tubes and 134 μL of carbonate buffer (0.05 M; pH 10.2) to the blank tube; then 1,666 μL of carbonate buffer (0.05 M, pH 10.2) was added to all tubes. The reaction was triggered by adding 200 μL of adrenaline (0.3 mM) to each tube and homogenizing the mixture. The absorbance was recorded after 20 and 80 s at 480 nm. The specific activity of SOD was defined as the unit of SOD required to cause a 50% inhibition of the oxidation of adrenaline to adenochrome in 1 min.

### 7.4 Malondialdehyde assay

Malondialdehyde (MDA) formed during lipid peroxidation reacts with thiobarbituric acid (TBA) in an acidic and hot environment to form a pink complex that can be quantified using a spectrophotometer at 530 nm (49). In tube containing 250 μL of homogenate (sample tubes) and control tubes containing 250 μL of Tris–HCl buffer (50 mM; pH = 7.4). In each of the tubes, 125 μL of trichloroacetic acid (TCA 20%) and 250 μL of thiobarbituric acid (TBA 0.67%) were added. The tubes were heated to 90 °C in a water bath for 10 min. They were cooled with tap water and centrifuged at 3,000 rpm at room temperature for 15 min. The supernatants were pipetted and the absorbance was read at 530 nm against the blank. The MDA concentration was calculated using the molar extinction coefficient (*ε* = 15,600 mol^−1^.cm^−1^).

### 7.5 Nitrite oxide assay

In the presence of amino-4-benzene sulfonamide and N-(naphthyl-1)-diamino-1,2-ethane dichloride (N-1-naphthyl ethylene diamine) in an acidic medium, nitrites undergo a diazotization reaction. The product obtained is proportional to the amount of nitrite present in the sample (53). Nitrite activity was measured using (53). In 100 μL of homogenate, 400 μL of distilled water was added, followed by 500 μL of Griess reagent followed by 200 μL of 1% sulfanilamide (5% orthophosphoric acid) and finally 200 μL of naphthyl ethylenediamine (1% NED prepared in tris-hydroxyl methylamine) The tubes were mixed and left in the dark for 5 min. The optical densities were read at 546 nm using a spectrophotometer. The nitric oxide level was determined by comparison with the standard sodium nitrite curve (0–100 μM) and expressed in μmol/mg of tissue.

## 8 Histological sections of brain structures

The whole brains were placed in plastic boxes containing formaldehyde (10%) and stored at room temperature. Each brain was sectioned into 100 μm thick slices of HPC, PFC, and ST, and the organ fragments were placed in numbered cassettes. After staining, the slides were dehydrated in three baths of 100% ethanol and then cleared in three baths of xylene (5 min per bath). After removal from the xylene, a few drops of resin were placed on the sections, which were then covered with a glass coverslip for observation at different magnifications (25×, 40×, 100×, 200×, 400×) under a microscope (Scientico STM-50) equipped with a microphotography device (Digital Microscope Suit 2.0 software) fitted with a Celestron 44421 digital camera connected to a computer.

## 9 Cell counting

Histomorphometry of the hippocampus, prefrontal cortex, and striatum was performed using Image J software. Counting is based on the reproducible identification and quantification of cells. The image is converted to 8-bit, and regions of interest are defined in order to maintain analysis on relevant areas. The image is then pre-processed by thresholding to convert the image to binary, isolating the cells from the background using the Otsu or Triangle method. Stuck cells are separated using the Watershed function, and counting is then performed using Analyze Particles, defining appropriate size and circularity criteria. The results (number of cells, area, circularity) are exported in CSV format, and the processing parameters are retained to ensure the comparability and reproducibility of the analyses between different images or experimental series (54).

## 10 Statistical analyses

Behavioral and biochemical data were processed using Microsoft^®^ Office Excel 2010 and graphs were constructed using Graph Pad Prism software for Windows, version 8.0.1. The normality of all data was assessed using the Kolmogorov–Smirnov test and the homogeneity of variance was confirmed using Barllett’s test. The results obtained were expressed as mean ± standard error of the mean (S.E.M) and the values were compared using the two-way ANOVA analysis of variance test, followed by Bonferroni’s *post hoc* multiple comparison test. Values were considered significant at *p* < 0.05.

## 11 Results

### 11.1 Effects of aqueous lyophilisate of *Ficus mucuso* bark on schizophrenia in the open arena test

#### 11.1.1 Effects of aqueous lyophilisate of *Ficus mucuso* on the number of lines crossed

**Figure 2:**
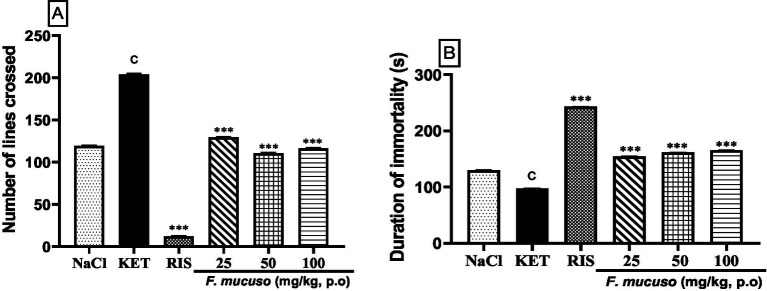
Effects of aqueous lyophilisate of *Ficus mucuso* on the number of lines crossed **(A)** and duration of immobility **(B)** in the open arena test. Each bar represents the mean ± SEM. *N* = 10; ^c^*p* < 0.001 compared to NaCl. ***p < 0.001 significant difference compared to KET. Two-way ANOVA followed by Bonferroni *post hoc* test. KET = Ketamine (20 mg/kg). NaCl = Saline solution (0.9%); RIS = Risperidone (0.5 mg/kg).

Figure 2 shows the curative effect of aqueous lyophilisate of *F. mucuso* on hyperlocomotion and immobility induced by chronic administration of ketamine as assessed in the open arena. The administration of ketamine (20 mg/kg, i.p.) for 14 days induced a significant increase (*p* < 0.001) in the number of lines crossed in mice, which rose from 119.5 ± 1.7 in the normal control (NaCl, 0.9%) to 204.0 ± 1.6 in the negative control group (KET). The aqueous lyophilisate (25, 50 and 100 mg/kg) caused a significant decrease [*F*(5, 54) = 9,545; *p* < 0.001] in the number of lines crossed to 129.3 ± 1.5; 110.5 ± 1.1, and 161.1 ± 1.9, respectively. Risperidone (0.5 mg/kg, p.o.) produced similar effects with a significant reduction (*p* < 0.001) in the number of lines crossed to 12.0 ± 1.6 compared to the negative control (Figure 2A).

#### 11.1.2 Effects of aqueous lyophilisate of *Ficus mucuso* on the duration of immobility induced

Figure 2B shows the effect of aqueous lyophilisate of *F. mucuso* on the duration of immobility induced by ketamine in the open arena. Chronic administration of ketamine (20 mg/kg, i.p.) induced a significant reduction (*p* < 0.001) in the duration of immobility. The duration of immobility decreased from 129.8 ± 1.88 s in the normal control group (NaCl, 0.9%) to 97.7 ± 1.17 s in the negative control group (KET). Administration of the aqueous lyophilisate of *F. mucuso* resulted in a significant increase [*F*(5, 54) = 5,649; *p* < 0.001] in the duration of immobility to 154.5 ± 1.4 s; 161.4 ± 1.52 and 165.5 ± 1.5 s at doses of 25, 50 and 100 mg/kg, respectively. Risperidone (0.5 mg/kg, p.o.) caused a significant increase (*p* < 0.001) in immobility time to 242.9 ± 1.48 s compared to the negative control (Figure 2B).

### 11.2 Effects of aqueous lyophilisate of *Ficus mucuso* bark on ketamine-induced schizophrenia in the Y-maze

#### 11.2.1 Effects of aqueous lyophilisate of *Ficus mucuso* on the number of spontaneous alternations

**Figure 3:**
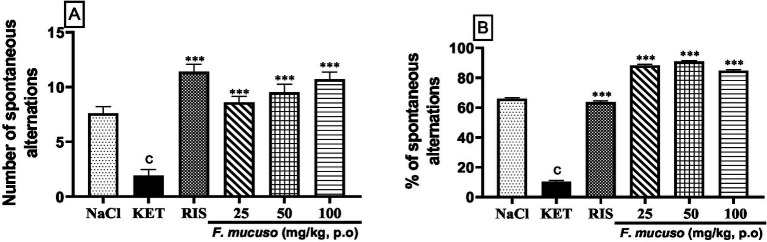
Effects of aqueous lyophilisate of *Ficus mucuso* on the number spontaneous alternations **(A)** and percentage of alternations **(B)** in the Y-maze test. Each bar represents the mean ± SEM. *N* = 10; ^c^*p* < 0.001 compared to NaCl. ***p < 0.001 significant difference compared to KET. Two-way ANOVA followed by Bonferroni post hoc test. KET = Ketamine (20 mg/kg). NaCl = Saline solution (0.9%); RIS = Risperidone (0.5 mg/kg).

Figure 3 shows that *F. mucuso* had a significant effect on the number and percentage of alternations in the Y-maze test. Chronic administration of ketamine (20 mg/kg, i.p.) for 14 days induced a significant decrease (*p* < 0.001) in the number of spontaneous alternations. This number decreased from 7.6 ± 1.6 in the normal control group (NaCl, 0.9%) to 1.9 ± 1.5 in the negative control group (KET). Administration of the aqueous lyophilisate of *F. mucuso* (25, 50, and 100 mg/kg) led to a significant increase [*F*(5, 54) = 28.8; *p* < 0.001] in the number of alternations to 8.6 ± 1.4, 9.5 ± 1.9, and 10.7 ± 1.84. Risperidone caused a significant increase (*p* < 0.001) of 11.4 ± 1.68 compared to the negative control (Figure 3A).

#### 11.2.2 Effects of the aqueous lyophilisate of *Ficus mucuso* on the percentage of alternations

Figure 3B shows the effect of the aqueous lyophilisate of *F. mucuso* on the percentage of alternation in the Y-maze test. Chronic administration of ketamine (20 mg/kg, i.p.) induced a significant decrease (*p* < 0.001) in the percentage of spontaneous alternations. This percentage decreased from 65.89 ± 1.94% in the normal group (NaCl, 0.9%) to 10.25 ± 1.86% in the negative control group (KET). The aqueous lyophilisate of *F. mucuso* (25, 50, and 100 mg/kg) caused a significant increase [*F*(5, 54) = 1,369; *p* < 0.001] in the percentage of alternations to 88.20 ± 1.76%; 90.76 ± 1.45, and 84.74 ± 68.01%. Risperidone caused a significant increase (*p* < 0.001) of 63.66 ± 1.90% compared to the negative control (Figure 3B).

### 11.3 Effects of aqueous lyophilisate of *Ficus mucuso* bark on schizophrenia induced by forced swimming

#### 11.3.1 Effects of aqueous lyophilisate *of Ficus mucuso* on swimming duration, immobility, and climbing time

**Figure 4:**
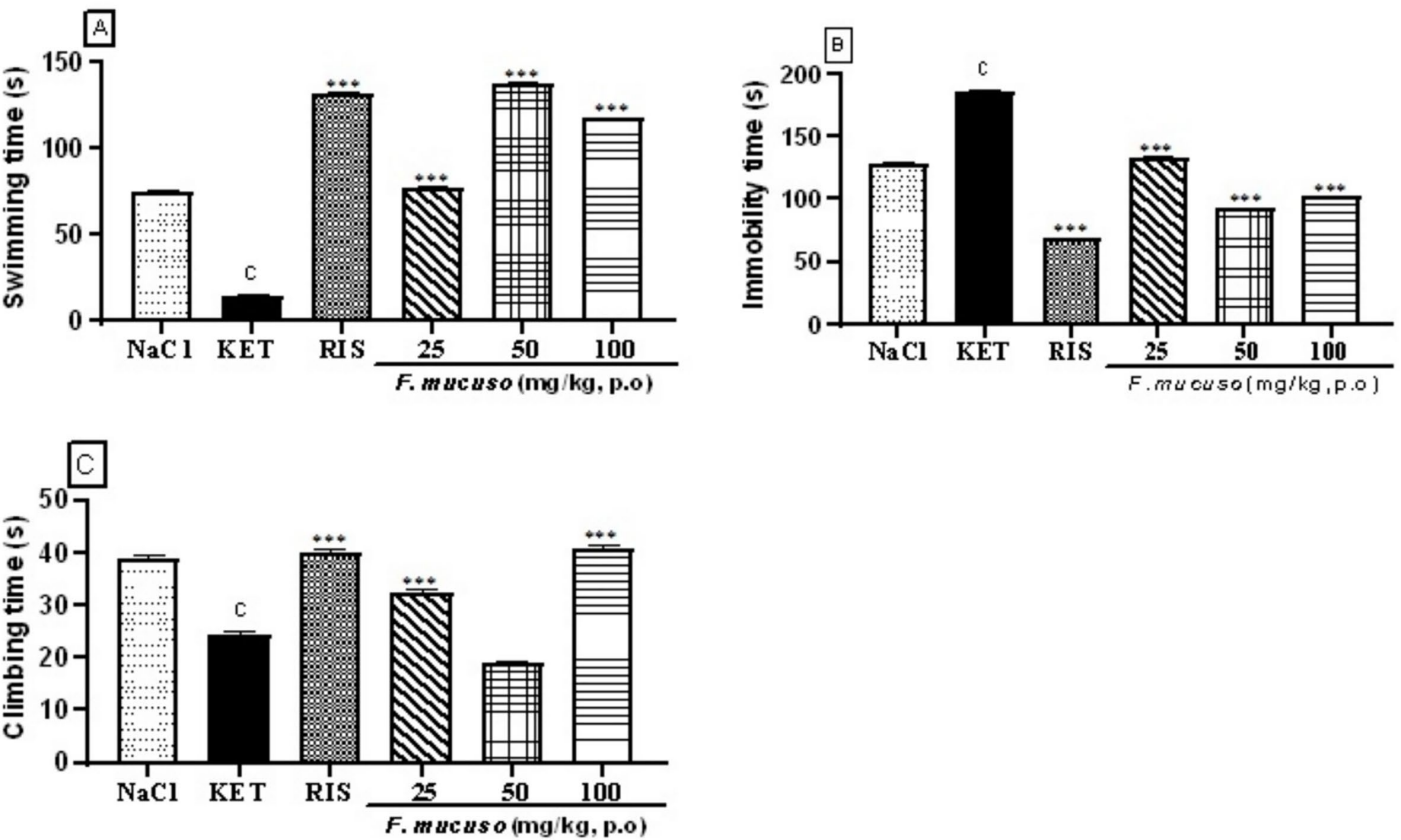
Effects of aqueous lyophilisate of *Ficus mucuso* on swimming time **(A)**, immobility time **(B)** and climbing duration **(C)** in the forced swim test. Each bar represents the mean ± SEM. *N* = 10; ^c^*p* < 0.001 compared to NaCl. ***p < 0.001 significant difference compared to KET. Two-way ANOVA followed by Bonferroni post hoc test. KET = Ketamine (20 mg/kg). NaCl = Saline solution (0.9%); RIS = Risperidone (0.5 mg/kg).

Figure 4 shows the effects of aqueous lyophilisate of *F. mucuso* on swimming time (Figure 4A), immobility time (Figure 4B), and climbing time (Figure 4C) induced by ketamine (20 mg/kg, i.p.) in the forced swimming test. Chronic administration of ketamine (20 mg/kg, i.p.) for 14 days caused a significant reduction (*p* < 0.001) in swimming time. Swimming duration decreased from 75.0 ± 1.8 s in the normal control group (NaCl, 0.9%) to 15.0 ± 1.4 s in the negative control group (KET). Administration of the aqueous lyophilisate (25, 50, and 100 mg/kg) led to a significant increase [*F*(5, 54) = 5,151; *p* < 0.001] in swimming time to 77.2 ± 1.64 s, 137.7 ± 1.5 s, and 117.6 ± 1.6 s. Risperidone (0.5 mg/kg) caused a significant increase (*p* < 0.001) in swimming time to 131.7 ± 1.84 s compared to the negative control (Figure 4A).

#### 11.3.2 Effects of the aqueous lyophilisate of *Ficus mucuso* on the duration of immobility

Figure 4B shows the effect of the aqueous lyophilisate of *F. mucuso* on the duration of immobility induced by ketamine (20 mg/kg) in the forced swim test. Chronic administration of ketamine (20 mg/kg, i.p.) induced a significant increase (*p* < 0.001) in the duration of immobility. This duration increased from 127.9 ± 1.72 s in the normal control group (NaCl, 0.9%) to 185.6 ± 1.52 s in the negative control group (KET). Administration of the aqueous lyophilisate (25, 50, and 100 mg/kg) significantly reduced [*F*(5, 54) = 3,188; *p* < 0.001] the duration of immobility to 133.1 ± 1.9 s; 92.8 ± 1.44 and 101.7 ± 1.96 s, respectively. Similar, risperidone caused a significant reduction (*p* < 0.001) in immobility duration to 39.6 ± 1.04 s compared to the negative control (Figure 4B).

#### 11.3.3 Effects of aqueous lyophilisate of *Ficus mucuso* on climbing duration

Figure 4C shows that the aqueous lyophilisate of *F. mucuso* had a significant effect on climbing duration in the forced swim test. Chronic administration of ketamine (20 mg/kg, i.p.) induced a significant decrease (*p* < 0.001) in climbing time. This time decreased from 38.8 ± 1.84 s in the normal control group (NaCl, 0.9%) to 24.4 ± 1.48 s in the negative control group (KET) group. The aqueous lyophilisate (25 and 100 mg/kg) caused a significant increase [*F*(5, 54) = 169; *p* < 0.001] in the climbing time to 32.3 ± 1.9 and 40.7 ± 1.96 s. Risperidone caused a significant increase (*p* < 0.001) to 39.9 ± 1.92 s in the climbing time compared to the negative control (Figure 4C).

### 11.4 Effects of aqueous lyophilisate of *Ficus mucuso* bark on schizophrenia induced in the stereotyped climbing test

#### 11.4.1 Effects of aqueous lyophilisate of *F. Mucuso* on climbing duration

**Figure 5:**
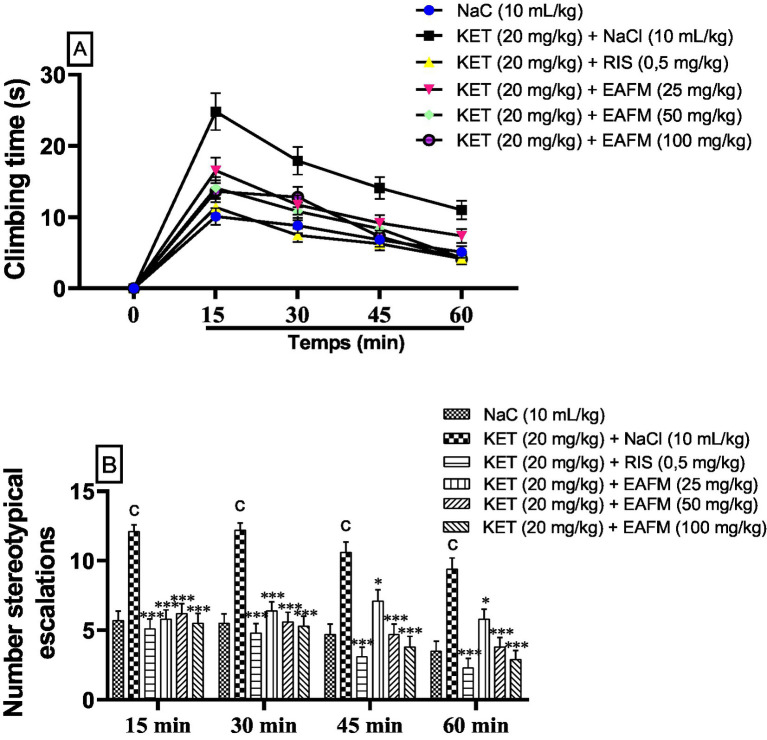
Effects of aqueous lyophilisate of *Ficus mucuso* on the climbing time **(A)** and number of stereotypical climbing **(B)** in the climbing test. Each bar represents the mean ± SEM. *N* = 10; ^c^*p* < 0.001 compared to NaCl. *p < 0.05; **p < 0.01; **p < 0.01; ***p < 0.001 significant difference compared to KET. Two-way ANOVA followed by Bonferroni post hoc test. KET = Ketamine (20 mg/kg). NaCl = Saline solution (0.9%); RIS = Risperidone (0.5 mg/kg).

Figure 5 shows that the aqueous lyophilisate of *F. mucuso* had a significant effect on the number (Figure 5A) and duration of climbing (Figure 5B) in the stereotyped climbing test. Chronic administration of ketamine (20 mg/kg, i.p.) for 14 days induced a significant increase (*p* < 0.001) in climbing duration. It increased from 11.1 ± 1.48 s; 9.7 ± 1.56; 7.5 ± 1.9 and 5.6 ± 1.68 s in the normal control group (NaCl, 0.9%) to 27.3 ± 1.96 s; 19.7 ± 1.82; 15.5 ± 1.9, and 12.1 ± 1.92 s in the negative control group (KET) respectively at 15, 30, 45, and 60 min after treatment. Figure 5A shows that the aqueous lyophilisate of *F. mucuso* (25, 50 and 100 mg/kg) caused a significant reduction [*F*(5, 54) = 63.0; *p* < 0.001] in stereotypical climbing time to 18.2 ± 1.84 s; 15.5 ± 1.8 s and 15.0 ± 1.8 s during the first 15 min and [*F*(5, 54) = 33.8; *p* < 0.001] in climbing time to 12.9 ± 1.92 s; 11.9 ± 1.72 and 14.1 ± 1.5 s at 30 min, followed by [*F*(5, 24) = 21.3; *p* < 0.001] to 10.1 ± 1.5 s; 9.2 ± 1.6 and 8.0 ± 1.6 s at 45 min and finally a decrease of (*p* < 0.01 and *p* < 0.001) [*F*(5, 54) = 16.7; *p* < 0.001] of 8.1 ± 1.72 s; 4.7 ± 2.1 and 4.5 ± 1.6 s with doses of 25, 50 and 100 mg/kg at 60 min. Risperidone caused a significant decrease (*p* < 0.001) in the duration of climbing to 12.5 ± 1.84 s; 8.2 ± 1.64; 6.9 ± 1.92, and 4.6 ± 1.92 s compared to the negative control (KET) at 15, 30, 45, and 60 min, respectively (Figure 5A).

#### 11.4.2 Effects of the aqueous lyophilisate of *Ficus mucuso* on the number of climbs

Figure 5B illustrates the significant effect of *F. mucuso* on the number of climbs in the stereotyped climbing test. Chronic administration of ketamine (20 mg/kg, i.p.) induced a significant increase (*p* < 0.001) in the number of climbs. The number of climbs increased from 5.7 ± 1.9; 5.5 ± 1.9; 4.7 ± 1.76 and 3.5 ± 1.9 in the normal control group (NaCl, 0.9%) to 12.1 ± 1.3; 12.2 ± 1.24; 10.6 ± 1.88 and 9.4 ± 1.888 in the negative control group (KET) respectively at 15, 30, 45, and 60 min after the last treatment. Figure 5C shows that the aqueous lyophilisate of *F. mucuso* (25, 50 and 100 mg/kg) significantly reduced [*F*(5, 54) = 16.1; *p* < 0.001] the number of stereotypical climbs to 5.8 ± 1.8; 6.2 ± 1.8 and 5.5 ± 1.76 during the first 15 min and [*F*(5, 54) = 17.7; *p* < 0.001] to 6.4 ± 1.6; 5.6 ± 1.92 and 5.3 ± 1.76 at 30 min there after (*p* < 0.05) [*F*(5, 24) = 13.8; *p* < 0.001] to 7.1 ± 1.92; 4.7 ± 1.96 and 3.8 ± 1.8 at 45 min and finally a decrease of [*F*(5, 54) = 13.8; *p* < 0.001] from 5.8 ± 1.84; 3.8 ± 1.8 and 2.9 ± 1.5 with doses of 25 mg/kg (*p* < 0.05); 50 and 100 mg/kg at 60 min. Risperidone caused a significant decrease (*p* < 0.001) in the number of escalations to 5.1 ± 1.92; 4.8 ± 1.8; 3.1 ± 1.72 and 2.3 ± 1.76 compared to the negative control (KET) at 15, 30, 45 and 60 min, respectively (Figure 5B).

### 11.5 Effects of aqueous lyophilisate of *Ficus mucuso* on neurotransmitter concentration

#### 11.5.1 Effects of aqueous lyophilisate of *Ficus mucuso* on dopamine concentration

**Figure 6:**
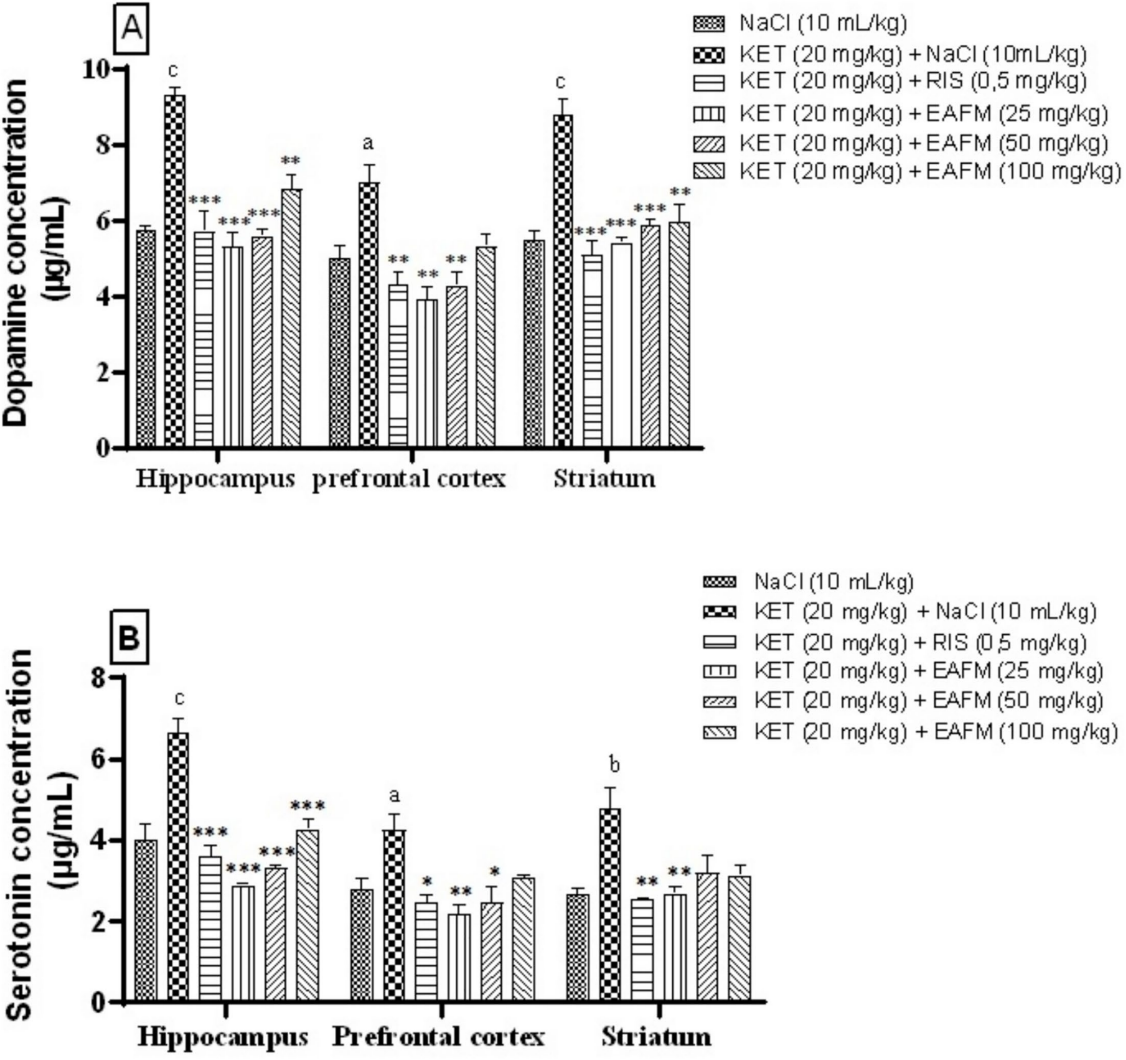
Effects of *Ficus mucuso* lyophilisate on dopamine **(A)** and serotonin **(B)** concentrations in the hippocampus, prefrontal cortex, and striatum of mice. Each bar represents the mean ± SEM. *N* = 10; ^a^*p* < 0.05; ^b^*p* < 0.01; ^c^*p* < 0.001 compared to NaCl. *p < 0.05; **p < 0.01; ***p < 0.001 significant difference compared to KET. Two-way ANOVA followed by Bonferroni post hoc test. KET = Ketamine (20 mg/kg). NaCl = Saline solution (0.9%); RIS = Risperidone (0.5 mg/kg).

Figure 6 shows that the aqueous lyophilisate of *F. mucuso* had a significant effect on dopamine concentration in the hippocampus, prefrontal cortex, and striatum. Administration of ketamine (20 mg/kg, i.p.) led to a significant increase (*p* < 0.05; *p* < 0.01 and *p* < 0.001) in dopamine concentration, which increased from 5.73 ± 0.03; 5.01 ± 0.10 and 5.47 ± 0.07 μg/mL in the normal control group to 9.28 ± 0.07; 7.01 ± 0.15 and 8.76 ± 0.13 μg/mL in the negative control group in the HPC, PFC, and ST, respectively. Administration of the aqueous lyophilisate of *F. mucuso* (Figure 6A) significantly decreased [*F*(5, 12) = 19.9; *p* < 0.001] the concentration of dopamine to 5.29 ± 0.12; 5.58 ± 0.05 and 6.83 ± 0.12 μg/mL with doses of 25, 50 and 100 mg/kg, respectively, in the HPC and [*F*(5, 12) = 9.01; *p* < 0.001] to 3.90 ± 0.10 and 4.27 ± 0.12 mM/min/g in the PFC for doses of 25 and 50 mg/kg (*p* < 0.01) of *F. mucuso* and finally [*F*(5, 12) = 15.3; *p* < 0.001] to 5.41 ± 0.04; 5.86 ± 0.06 and 5.94 ± 0.15 μg/mL in the ST compared to the negative control (KET). The significant increase (*p* < 0.01 and *p* < 0.001) in the amount of dopamine to 5.72 ± 0.17; 4.30 ± 0.11 and 5.08 ± 0.11 μg/mL with Risperidone (0.5 mg/kg, i.p.) in the PFC, HPC and ST compared to the negative control (KET).

#### 11.5.2 Effects of aqueous lyophilisate of *Ficus mucuso* on serotonin concentration

The cerebral serotonin concentration is shown in Figure 6B. Administration of ketamine (20 mg/kg, i.p.) caused a significant increase (*p* < 0.001; *p* < 0.01 and *p* < 0.05) in serotonin concentration in the HPC, PFC and ST, respectively. This concentration increased from 3.99 ± 0.13, 3.11 ± 0.09, and 2.67 ± 0.04 μg/mL in the normal control to 6.63 ± 0.12, 4.24 ± 0.11, and 4.77 ± 0.17 μg/mL in the negative control. The aqueous lyophilisate of *F. mucuso* (25, 50 and 100 mg/kg) significantly decreased (*p* < 0.01; *p* < 0.001) [*F*(5, 12) = 22.4; *p* < 0.001] the concentration of 5-TH2 to 2.88 ± 0.01; 3.32 ± 0.02 and 4.26 ± 0.08 mol/g in the HPC; [*F*(5, 12) = 7.85; *p* = 0.002] to 2.18 ± 0.06 and 2.44 ± 0.13 μg/mL in the PFC with the 25 mg/kg (*p* < 0.01) and 50 mg/kg (*p* < 0.05) doses; finally [*F*(5, 12) = 2.68; *p* = 0.003] to 2.68 ± 0.05 μg/mL with the 25 mg/kg dose (*p* < 0.01) in the ST compared to the negative control (KET). Risperidone (0.5 mg/kg) caused a significant decrease (*p* < 0.001) in serotonin concentration to 3.58 ± 0.09; 2.45 ± 0.06 and 2.52 ± 0.01 μg/mL in the brain regions, respectively, (HPC, PFC and ST).

#### 11.5.3 Effects of aqueous lyophilisate of *Ficus mucuso* on cerebral glutamate levels

**Figure 7:**
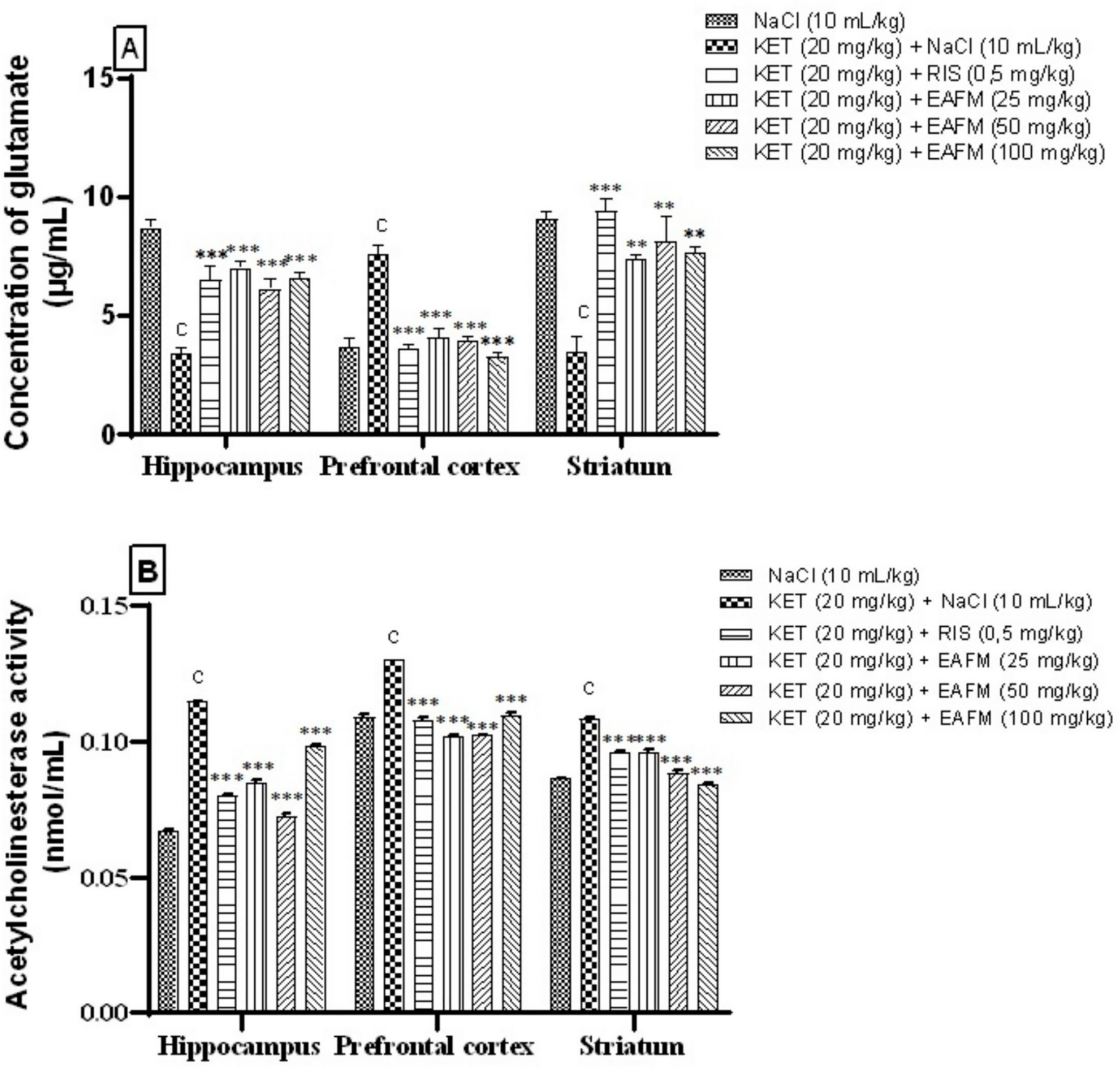
Effects of *Ficus mucuso* lyophilisate on glutamate levels **(A)** and acetylcholinesterase activity **(B)** in the hippocampus, prefrontal cortex, and striatum of mice. Each bar represents the mean ± SEM. *N* = 10; ^c^*p* < 0.001 compared to NaCl. **p < 0.01; ***p < 0.001 significant difference compared to KET. Two-way ANOVA followed by Bonferroni post hoc test. KET = Ketamine (20 mg/kg). NaCl = Saline solution (0.9%); RIS = Risperidone (0.5 mg/kg).

Figure 7 shows that aqueous lyophilisate of *F. mucuso* had a significant effect on glutamate levels in the hippocampus, prefrontal cortex, and striatum. Figure 7A showed that administration of ketamine (20 mg/kg, i.p.) resulted in a significant decrease (*p* < 0.001) in glutamate levels, which fell from 3.67 ± 0.16 and 9.07 ± 0.15 μg/mL in the normal control group to 3.37 ± 0.12 and 3.48 ± 0.31 μg/mL in the negative control (KET) group in the HPC and ST, respectively; However, glutamate increased significantly (*p* < 0.001) in the PFC, rising from 3.69 ± 0.16 μg/mL in the normal control group to 7.54 ± 0.19 μg/mL in the negative control group. However, administration of the aqueous lyophilisate of *F. mucuso* (Figure 7A) significantly increased [*F*(5, 12) = 33.0; *p* < 0.001] the glutamate level to 6.99 ± 0.13; 6.10 ± 0.21 and 6.57 ± 0.10 μg/mL at all doses, respectively, in the HPC and [*F*(5, 12) = 12.7; *p* < 0.001] to 7.38 ± 0.24; 8.11 ± 0.51 and 7.64 ± 0.12 μg/mL in the ST at doses of 25, 50 and 100 mg/kg (*p* < 0.01). In contrast, the opposite effect was observed in the PFC, with a significant decrease [*F*(5, 12) = 26.5; *p* < 0.001] to 4.07 ± 0.19; 3.93 ± 0.09 and 3.27 ± 0.07 μg/mL, respectively. However, a significant increase (*p* < 0.001) in glutamate levels to 6.47 ± 0.01 and 9.38 ± 0.24 in the HPC and ST, respectively and a decrease (*p* < 0.001) to 3.59 ± 0.08 μg/mL in the PFC was observed with risperidone (0.5 mg/kg) compared to the negative control.

#### 11.5.4 Effects of aqueous lyophilisate of *Ficus mucuso* on acetylcholinesterase activity

Analysis of Figure 7B reveals that ketamine administration led to a significant increase (*p* < 0.001) in acetylcholinesterase activity, which rose from 0.06 ± 0.00 nm/mL; 0.10 ± 0.00 and 0.08 ± 0.00 nm/mL in the normal control group to 0.11 ± 0.00; 0.13 ± 0.00 and 0.10 ± 0.00 nm/mL in the negative control (KET) respectively in the hippocampus, prefrontal cortex, and striatum. Administration of the aqueous lyophilisate of *F. mucuso* (Figure 7B) significantly decreased [*F*(5, 12) = 1,512, *p* < 0.001] acetylcholinesterase activity to 0.08 ± 0.00; 0.07 ± 0.00 and 0.95 ± 0.09 nm/mL with doses of 25, 50, and 100 mg/kg, respectively, in the HPC, and a decrease [*F*(5, 12) = 379, *p* < 0.001] to 0.10 ± 0.00; 0.10 ± 0.00 and 0.10 ± 0.00 nm/mL in the PFC and finally a significant decrease [*F*(5, 12) = 258, *p* < 0.001] to 0.09 ± 0.00; 0.08 ± 0.00 and 0.08 ± 0.00 nm/mL in the ST compared to the negative control. A decrease in acetylcholinesterase activity was also observed with risperidone (0.5 mg/kg, i.p.) at 0.08 ± 0.00; 0.10 ± 0.00 and 0.09 ± 0.00 nm/mL in the HPC, PFC and ST, respectively.

#### 11.5.5 Effects of aqueous lyophilisate of *Ficus mucuso* on the concentration of gamma-aminobutyric acid in the brain

**Figure 8:**
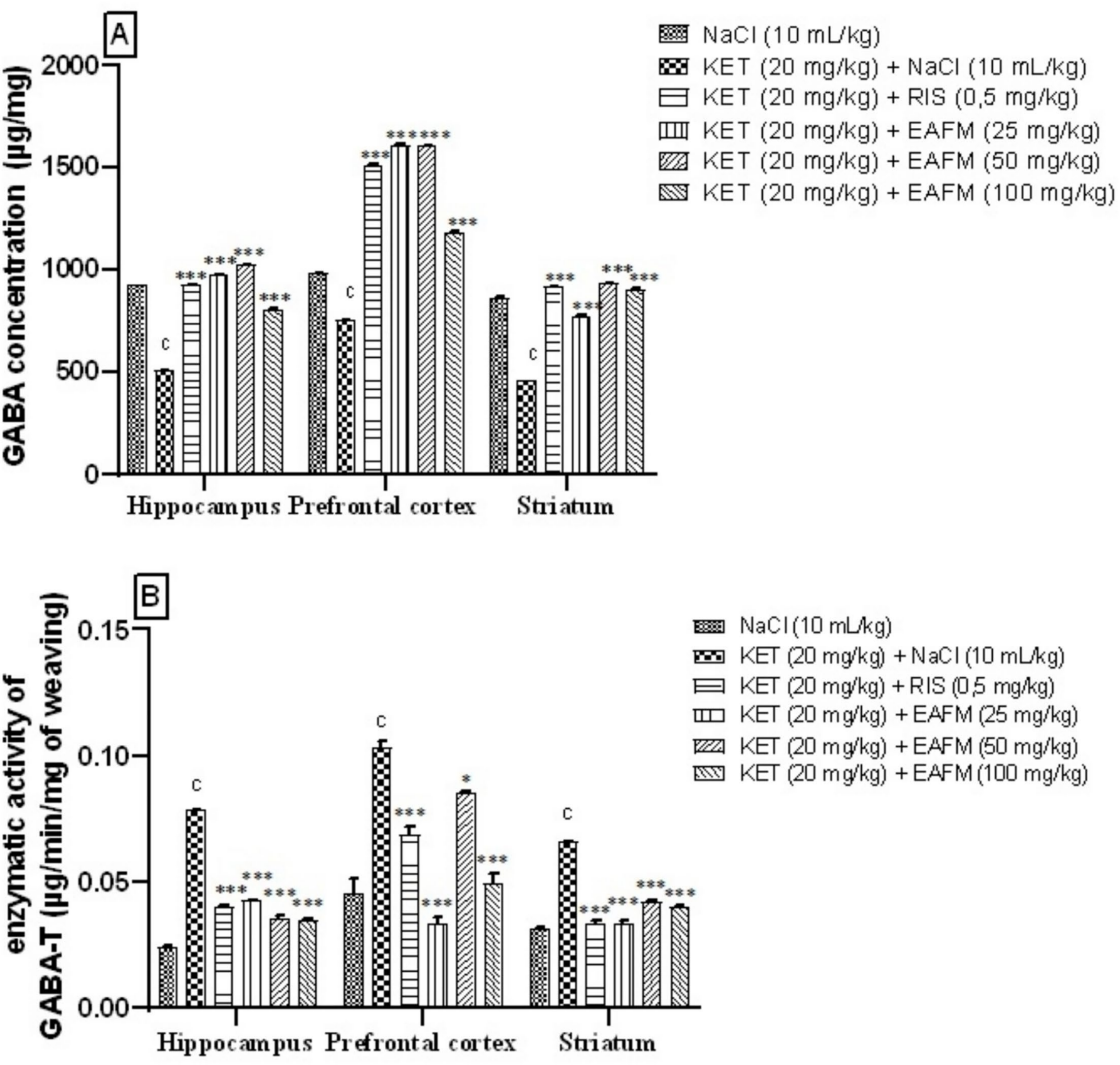
Effects of *Ficus mucuso* lyophilisate on gamma-aminobutyric acid **(A)** concentration and gamma-aminobutyric acid transaminase activity **(B)** in the hippocampus, prefrontal cortex, and striatum of mice. Each bar represents the mean ± SEM. *N* = 10; ^c^*p* < 0.001 compared to NaCl. ***p < 0.001 significant difference compared to KET. Two-way ANOVA followed by Bonferroni post hoc test. KET = Ketamine (20 mg/kg). NaCl = Saline solution (0.9%); RIS = Risperidone (0.5 mg/kg).

Figure 8A shows the concentration of GABA in the hippocampus, prefrontal cortex, and striatum. Administration of ketamine (20 mg/kg, i.p.) for 14 days significantly decreased (*p* < 0.001) the concentration of GABA in the HPC, PFC, and ST. The concentration of GABA decreased from 926.36 ± 0.12 μg/mg; 982.38 ± 0.50, and 864.83 ± 0.55 μg/mg in the normal control to 507.56 ± 0.24; 753.56 ± 0.52, and 461.65 ± 0.11 μg/mg in the negative control. Figure 8A shows that the aqueous lyophilisate of *F. mucuso* (25, 50, and 100 mg/kg) significantly increased [*F*(5, 12) = 26,996; *p* < 0.001] the concentration of GABA, which rose to 973.44 ± 0.55 μg/mg; 1,023.49 ± 0.40 and 807.34 ± 0.19 μg/mg in HPC; [*F*(5, 12) = 73,173; *p* < 0.001] to 1,609.94 ± 0.24 μg/mg; 1,607.31 ± 0.44 and 1,182.45 ± 0.40 μg/mg in the PFC and finally [*F*(5, 12) = 14,690; *p* < 0.001] at 772.28 ± 0.66 μg/mg; 933.80 ± 0.31 and 904.74 ± 0.26 μg/mg in the ST compared to the negative control (KET). Risperidone (0.5 mg/kg) had a similar effect, significantly increasing (*p* < 0.001) the concentration of GABA to 927.36 ± 0.39; 1,512.78 ± 0.35 and 917.54 ± 0.58 μg/mg in the HPC, PFC, and ST (Figure 8A).

#### 11.5.6 Effects of aqueous lyophilisate of *Ficus mucuso* on the concentration of gamma-aminobutyric acid transferase in the brain

Figure 8B highlights the activity of the enzyme concentration of GABA-T in the hippocampus, prefrontal cortex, and striatum. The administration of ketamine (20 mg/kg, i.p.) significantly increased (*p* < 0.001) the enzymatic activity of GABA-T in the HPC, PFC, and ST. GABA-T activity increased from 0.02 ± 0.00 μg/min/mg; 0.04 ± 0.00 and 0.03 ± 0.00 μg/min/mg in the normal control to 0.07 ± 0.00; 0.10 ± 0.00 and 0.06 ± 0.00 μg/min/mg in the negative control (KET). Figure 8B shows that the aqueous lyophilisate of *F. mucuso* (25, 50 and 100 mg/kg) significantly decreased [*F*(5, 12) = 1,186; *p* < 0.001] the activity of GABA-T, which fell to 0.04 ± 0.00 μg/min/mg; 0.03 ± 0.00 and 0.03 ± 0.00 μg/min/mg in the HPC; [*F*(5, 12) = 56.5; *p* < 0.001] to 0.03 ± 0.00 μg/min/mg; 0.08 ± 0.00 and 0.04 ± 0.00 μg/min/mg in the PFC and finally [*F*(5, 12) = 270; *p* < 0.001] to 0.03 ± 0.00 μg/min/mg; 0.04 ± 0.00 and 0.04 ± 0.00 μg/min/mg in the ST compared to the negative control. Risperidone (0.5 mg/kg) significantly decreased (*p* < 0.001) GABA-T enzyme activity to 0.04 ± 0.00; 0.06 ± 0.00 and 0.03 ± 0.00 μg/min/mg in the HPC, PFC and ST, respectively (Figure 8B).

### 11.6 Effects of aqueous lyophilisate of *Ficus mucuso* bark on markers of oxidative stress in the brain

#### 11.6.1 Effects of aqueous lyophilisate of *Ficus mucuso* on catalase activity

**Figure 9:**
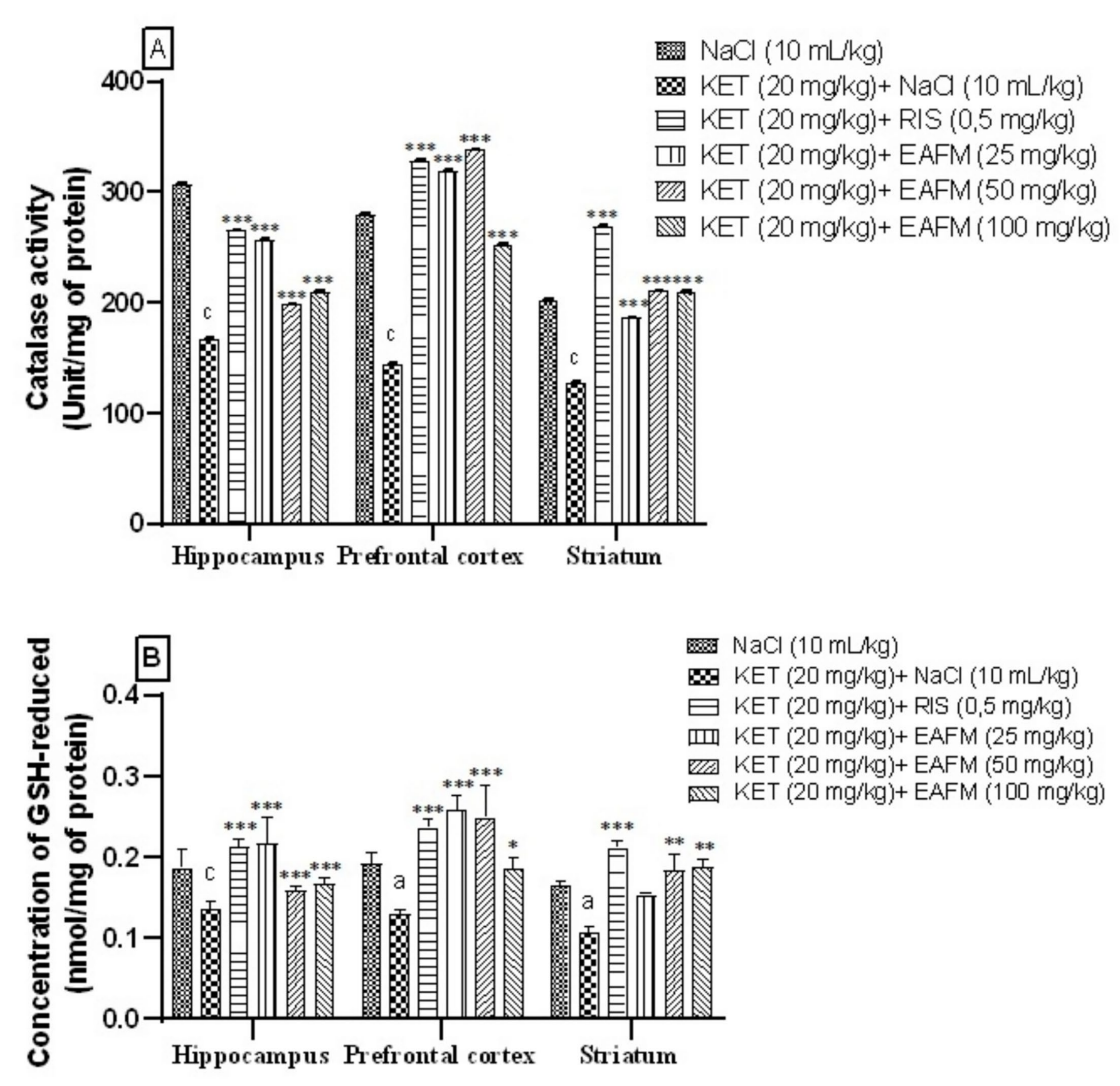
Effects of *Ficus mucuso* lyophilisate on catalase activity **(A)** and reduced glutathione **(B)** concentration in the hippocampus, prefrontal cortex, and striatum of mice. Each bar represents the mean ± SEM. *N* = 10; ^a^*p* < 0.05; ^c^*p* < 0.001 compared to NaCl. *p < 0.05; **p < 0.01; ***p < 0.001 significant difference compared to KET. Two-way ANOVA followed by Bonferroni post hoc test. KET = Ketamine (20 mg/kg). NaCl = Saline solution (0.9%); RIS = Risperidone (0.5 mg/kg).

Figure 9 shows the effect of aqueous lyophilisate of *F. mucuso* on catalase activity in the hippocampus, prefrontal cortex and striatum. Analysis of Figure 9A reveals that administration of ketamine (20 mg/kg, i.p.) caused a significant decrease (*p* < 0.001) in catalase activity, which fell from 306.68 ± 0.28; 279.27 ± 0.24 and 202.50 ± 0.23 nmol/unit/mg protein in the normal control group to 167.03 ± 0.19; 144.88 ± 0.31 and 128.12 ± 0.10 nmol/unit/mg protein in the negative control in the HPC, PFC and ST, respectively. Administration of the aqueous lyophilisate of *F. mucuso* (Figure 9A) significantly increased [*F*(5, 12) = 6,216, *p* < 0.001] CAT activity to 256.91 ± 0.79; 198.29 ± 0.54 and 209.95 ± 0.79 nmol/unit/mg protein with doses of 25, 50 and 100 mg/kg, respectively, in the HPC and [*F*(5, 12) = 10,706, *p* < 0.001] to 319.37 ± 0.14; 337.72 ± 0.12 and 253.25 ± 0.16 nmol/unit/mg protein in the PFC and finally an increase of [*F*(5, 12) = 7,435, *p* < 0.001] to 186.29 ± 0.12; 210.50 ± 0.05 and 209.94 ± 0.22 nmol/unit/mg protein in the ST compared to the negative control (KET). There was a significant increase (*p* < 0.001) in CAT activity to 265.29 ± 0.41; 328.13 ± 0.29 and 269.30 ± 0.20 nmol/unit/mg protein with Risperidone (0.5 mg/kg, i.p) in the PFC, HPC and ST (Figure 9A).

### 11.7 Effects of aqueous lyophilisate of *Ficus mucuso* on reduced glutathione concentration

Figure 9B shows the effect of aqueous lyophilisate of *F. mucuso* on reduced glutathione concentration in the hippocampus, prefrontal cortex, and striatum after chronic administration of ketamine (20 mg/kg, i.p.). Ketamine induced a significant decrease (*p* < 0.05) and (*p* < 0.001) in GSH levels in the brain regions. It decreased from 0.187 ± 0.010; 0.192 ± 0.006 and 0.165 ± 0.003 nmol/mg protein in the normal control (KET) to 0.136 ± 0.004; 0.129 ± 0.002 and 0.107 ± 0.003 nmol/mg protein in the negative control (KET) in the HPC, PFC, and ST, respectively. The GSH level increased significantly [*F*(5, 12) = 149, *p* < 0.001] to 0.216 ± 0.15; 0.159 ± 0.002 and 0.166 ± 0.003 nmol/mg protein in the HPC with doses of 25, 50, and 100 mg/kg; [*F*(5, 12) = 22.1, *p* < 0.001] to 0.258 ± 0.008; 0.249 ± 0.018 with doses of 25 and 50 mg/kg and finally 0.186 ± 0.006 with a dose of 100 mg/kg (*p* < 0.05) in PFC. Risperidone (0.5 mg/kg) significantly increased (*p* < 0.001) the cerebral GSH level to 0.212 ± 0.005; 0.237 ± 0.004 and 0.211 ± 0.004 nmol/mg protein, respectively, in the HPC, PFC and ST (Figure 9B).

#### 11.7.1 Effects of aqueous lyophilisate *Ficus mucuso* on superoxide dismutase concentration

**Figure 10:**
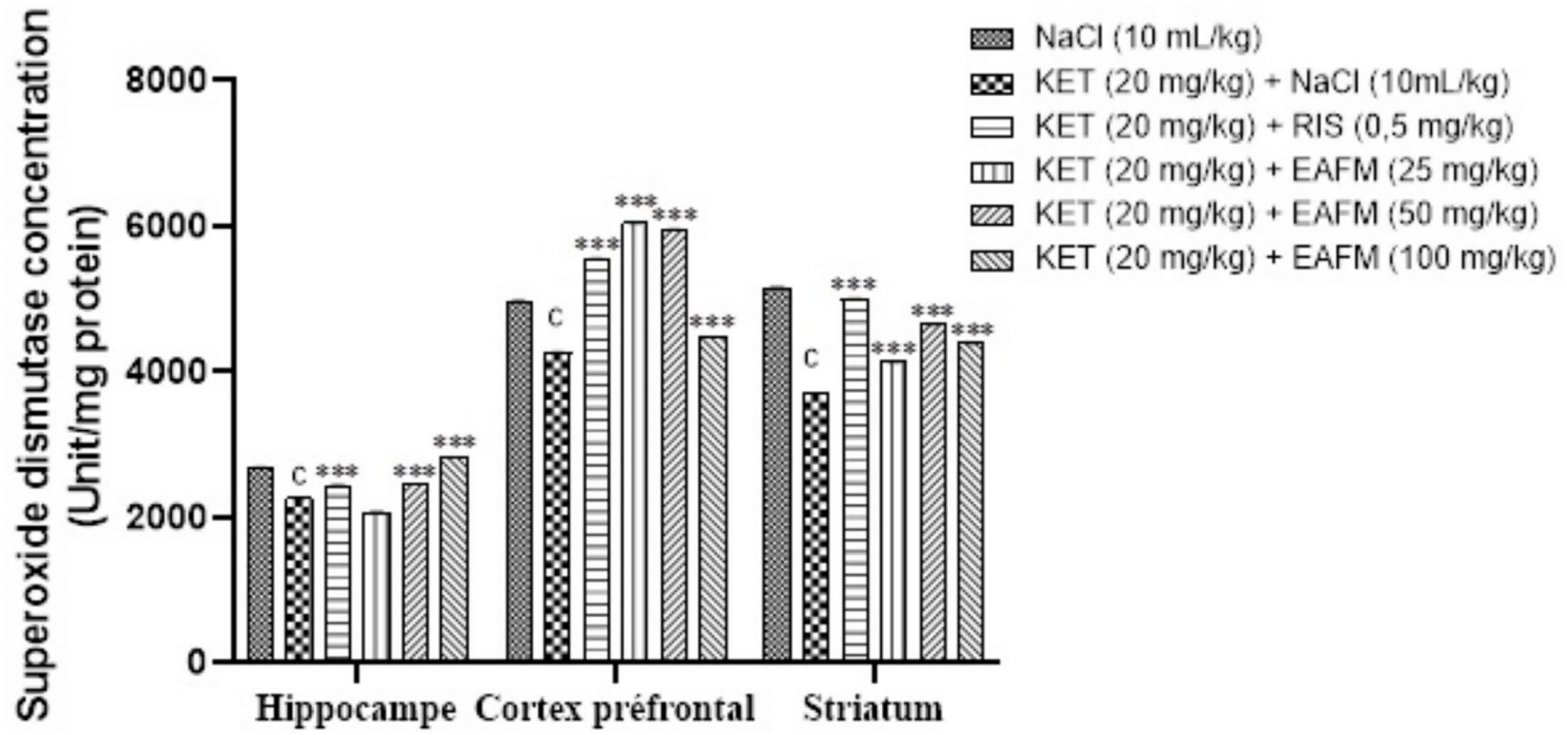
Effects of *Ficus mucuso* lyophilisate on superoxide dismutase concentration in the hippocampus, prefrontal cortex, and striatum of mice. Each bar represents the mean ± SEM. *N* = 10; *p* < 0.001 compared to NaCl. ***p < 0.001 significant difference compared to KET. Two-way ANOVA followed by Bonferroni post hoc test. KET = Ketamine (20 mg/kg). NaCl = Saline solution (0.9%); RIS = Risperidone (0.5 mg/kg).

The concentration of superoxide dismutase is shown in Figure 10. The administration of ketamine (20 mg/kg, i.p.) led to a significant decrease (*p* < 0.001) in SOD levels in the hippocampus, prefrontal cortex, and striatum. This concentration decreased from 2,677.41 ± 0.43 Unit/mg protein; 4,963.25 ± 0.56, and 5,152.43 ± 1.00 SOD units/mL/g in the normal control group to 2,259.19 ± 0.49; 4,256.39 ± 0.63, and 3,693.15 ± 0.85 Unit/mg protein in the negative control group. The aqueous lyophilisate of *F. mucuso* (50 and 100 mg/kg) significantly increased [*F*(5, 12) = 76,689] the SOD concentration to 2,473.87 ± 0.47 and 2,857.48 ± 0.45 Unit/mg protein in the HPC; an increase [*F*(5, 12) = 378,290, *p* < 0.001] to 6,041.96 ± 0.40; 5,945.22 ± 0.58 and 4,468.22 ± 0.60 Unit/mg protein in PFC; and finally an increase [*F*(5, 12) = 100,494, *p* < 0.001] to 4,134.40 ± 0.92; 4,647.40 ± 0.51 and 4,385.93 ± 0.73 Unit/mg protein in the ST compared to the negative control (KET). Risperidone (0.5 mg/kg) significantly increased (*p* < 0.001) the concentration of SOD to 2,435.65 ± 0.25; 5,543.41 ± 0.65 and 4,993.83 ± 0.75 Unit/mg protein in the HPC, PFC and ST (Figure 10).

#### 11.7.2 Effects of aqueous lyophilisate of *Ficus mucuso* on malondialdehyde concentration

**Figure 11:**
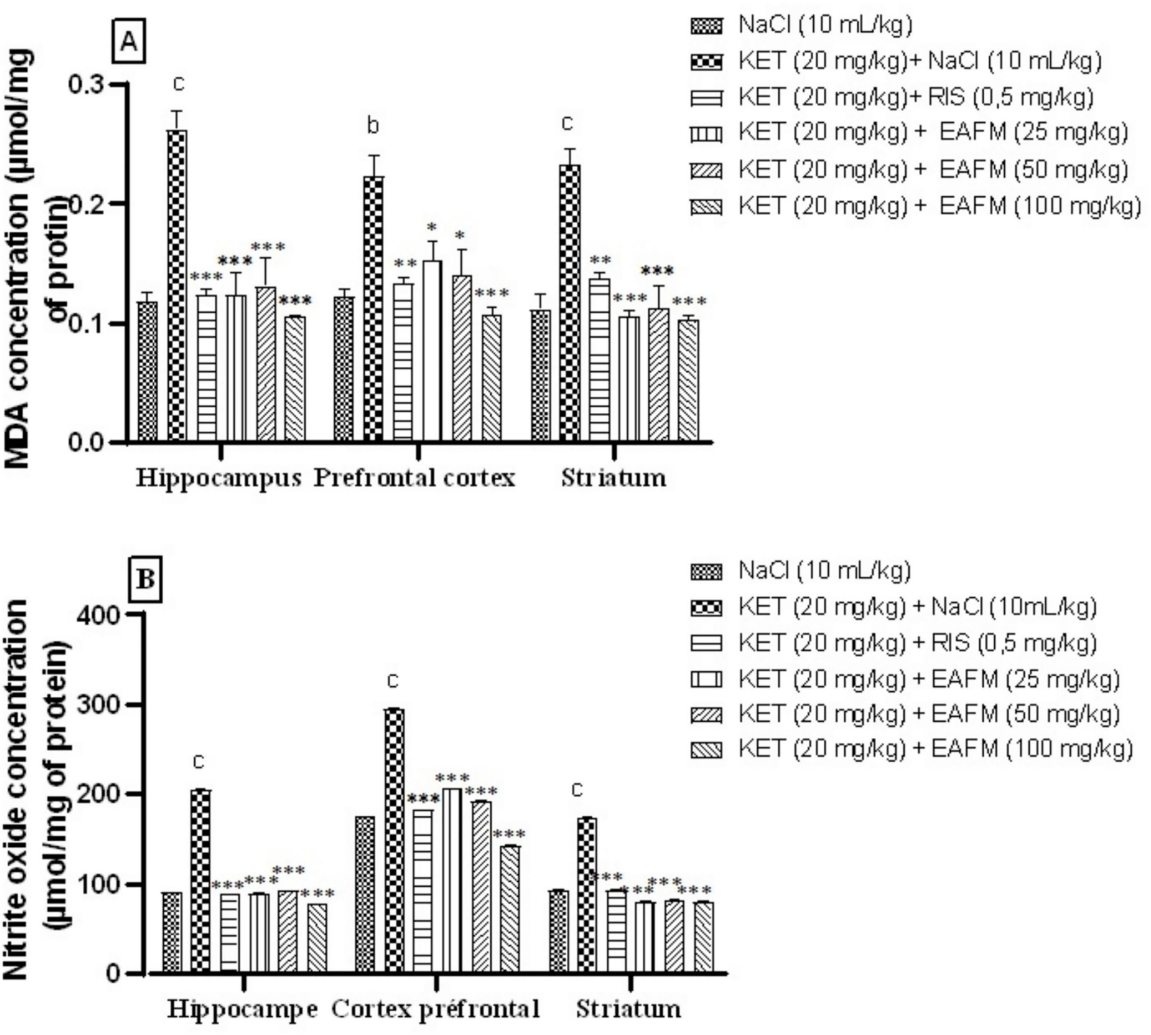
Effects of *Ficus mucuso* lyophilisate on malondialdehyde **(A)** and nitrite oxide **(B)** concentrations in the hippocampus, prefrontal cortex, and striatum of mice. Each bar represents the mean ± SEM. *N* = 10; ^b^*p* < 0.01; ^c^*p* < 0.001 compared to NaCl. *p < 0.05; **p < 0.001; ***p < 0.001 significant difference compared to KET. Two-way ANOVA followed by Bonferroni post hoc test. KET = Ketamine (20 mg/kg). NaCl = Saline solution (0.9%); RIS = Risperidone (0.5 mg/kg).

Figure 11 shows the effect of aqueous lyophilisate of *F. mucuso* on malondialdehyde concentration (Figure 11A). Ketamine (20 mg/kg) induced a significant increase (*p* < 0.01; *p* < 0.001) in malondialdehyde concentration to 0.262 ± 0.007 μmol/mg protein; 0.223 ± 0.008 and 0.232 ± 0.006 μmol/mg protein in the hippocampus, prefrontal cortex, and striatum, respectively, compared to the normal control of 0.117 ± 0.004 μmol/mg protein; 0.122 ± 0.003 and 0.111 ± 0.006 μmol/mg protein. Administration of the aqueous lyophilisate of *F. mucuso* (25; 50, and 100 mg/kg) resulted in a significant reduction [*F*(5, 12) = 18.7, *p* < 0.001] in MDA concentration to 0.126 ± 0.008; 0.131 ± 0.011, and 0.104 ± 0.000 in the HPC; (*p* < 0.05; *p* < 0.001) [*F*(5, 12) = 9.07, *p* < 0.001] to 0.152 ± 0.007; 0.139 ± 0.010 and 0.106 ± 0.009 μmol/mg protein in the PFC and finally a reduction (*p* < 0.001) [*F*(5, 12) = 18.8, *p* < 0.001] in the MDA level to 0.105 ± 0.005; 0.112 ± 0.009 and 0.102 ± 0.001 μmol/mg protein in ST compared to the negative control (KET). Risperidone (0.5 mg/kg, i.p.) induced a significant reduction (*p* < 0.001; *p* < 0.01) in MDA concentration to 0.123 ± 0.002; 0.132 ± 0.002 and 0.137 ± 0.002 mol/g in HPC, PFC and ST, respectively, compared to the negative control (Figure 11A).

#### 11.7.3 Effects of *Ficus mucuso* aqueous lyophilisate on cerebral nitrite oxide concentration

The nitrite concentration is shown in Figure 11B. The administration of ketamine (20 mg/kg, i.p.) led to a significant increase (*p* < 0.001) in NO levels in the hippocampus, prefrontal cortex, and striatum. This increase rose from 204.70 ± 0.11 μmol/mg protein; 295.23 ± 0.22 and 174.07 ± 0.52 μmol/mg protein in the normal control group to 90.10 ± 0.10 μmol/mg protein; 175.15 ± 0.12 and 93.55 ± 0.17 μmol/mg protein in the negative control group (KET). Administration of the aqueous lyophilisate (25, 50 and 100 mg/kg) resulted in a significant decrease [*F*(5, 12) = 21,029, *p* < 0.001] in NO concentration, which fell to 89.86 ± 0.31 μmol/mg protein; 92.82 ± 0.09 and 77.88 ± 0.11 μmol/mg protein in the HPC; a significant decrease [*F*(5, 12) = 9,648, *p* < 0.001] to 206.17 ± 0.23 mol/g; 192.58 ± 0.15 and 142.72 ± 0.21 mol/g in the PFC; and finally a significant decrease [*F*(5, 12) = 5,474, *p* < 0.001] to 80.40 ± 0.05; 81.62 ± 0.18 and 79.64 ± 0.17 μmol/mg protein in the striatum compared to the negative control (KET). Risperidone (0.5 mg/kg) caused a significant decrease (*p* < 0.001) in NO levels to 89.00 ± 0.11 μmol/mg protein; 182.80 ± 0.01 and 93.46 ± 0.16 μmol/mg protein, respectively, in the HPC, PFC, and ST (Figure 11B).

### 11.8 Effects of aqueous lyophilisate of *Ficus mucuso* bark on alterations in brain structures

#### 11.8.1 Effects of aqueous lyophilisate of *Ficus mucuso* on Ammon’s horn 1 & 3 and the dentate gyrus of the hippocampus

**Figure 12:**
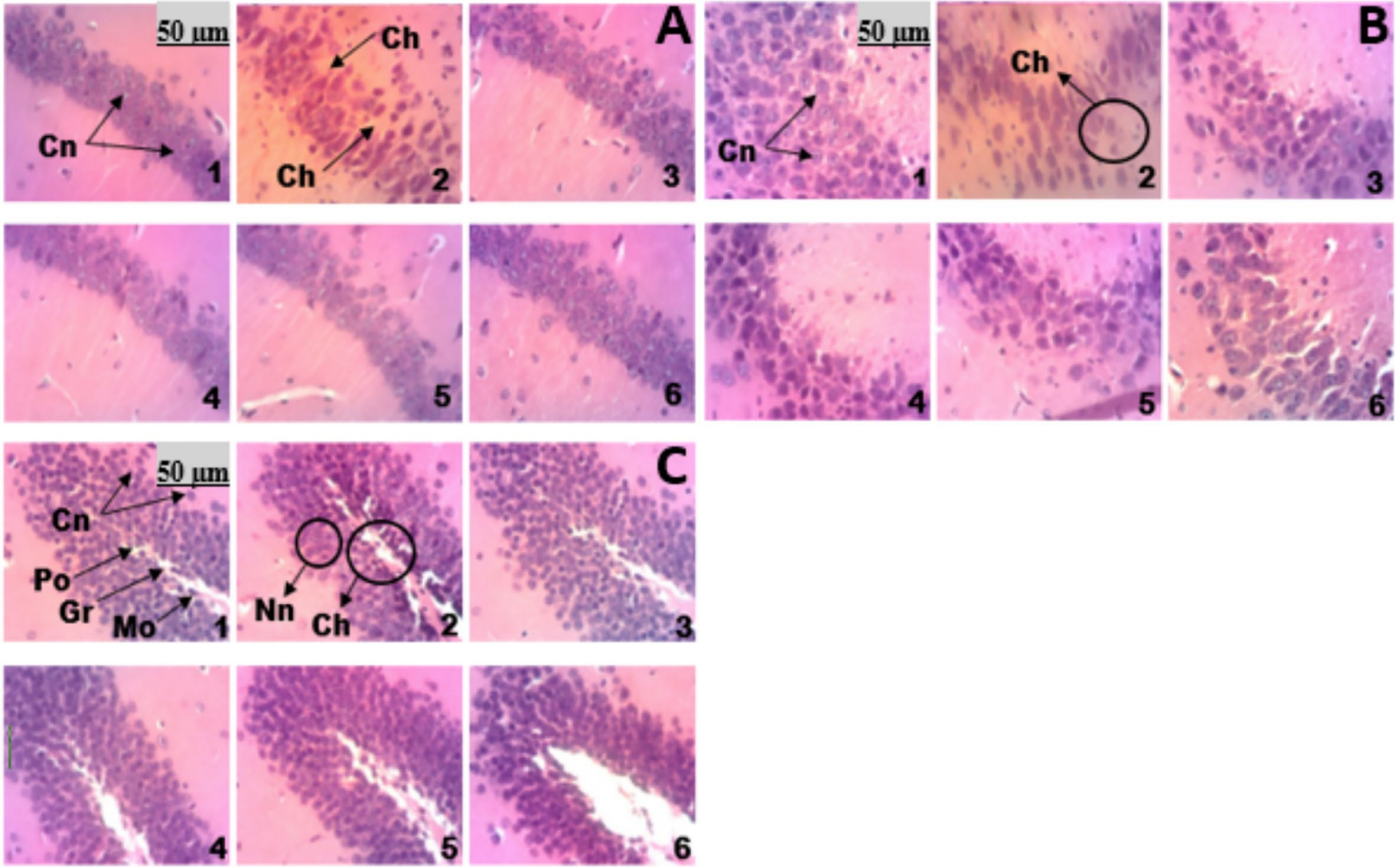
Microphotographs of the Ammon’s horn 1 & 3 and the dentate gyrus of the hippocampus (×250); hematoxylin–eosin staining. 1 = Normal control; 2 = Negative control; 3 = Positive control; 4–6 = Groups receiving the aqueous lyophilisate of *F. mucuso* (25, 50, and 100 mg/kg). Cn = Normal cells; Ch = Hyperchromatic cells; Nn = Necrotic neurons; Cn = Normal cells; Po = Polymorphic layer; Gr = Granular layer; Mo = Molecular layer; Po = Polymorphic layer; Gr = Granular layer; Mo = Molecular layer.

The histopathological changes induced by ketamine in the hippocampus (Figure 12) were revealed by hematoxylin–eosin staining. Histological analysis of Ammon’s horn 1 (Figure 12A), Ammon’s horn 3 (Figure 12B) and the dentate gyrus (Figure 12B) showed a normal microstructure in mice from the normal group (microphotograph 1). In mice from the negative control group (microphotograph 2), a loss of neuronal tissue integrity was observed, marked by the presence of hyperchromatic cells (Figures 12A,B), degradation of the polymorphic, granular, and molecular layers, and necrosis (Figure 12C). The groups treated with the aqueous lyophilisate at different doses of 25, 50 and 100 mg/kg (microphotographs 4, 5 and 6) and the group treated with risperidone (microphotograph 3) (Figures 12A–C) showed a microstructure of Ammon’s horn 1 & 3 and the dentate gyrus with a healthy neuronal architecture similar to that of the normal control (microphotograph 1).

#### 11.8.2 Effects of aqueous lyophilisate of *Ficus mucuso* on changes in cell numbers in Ammon’s horn 1 and 3 and the dentate gyrus of the hippocampus

**Figure 13:**
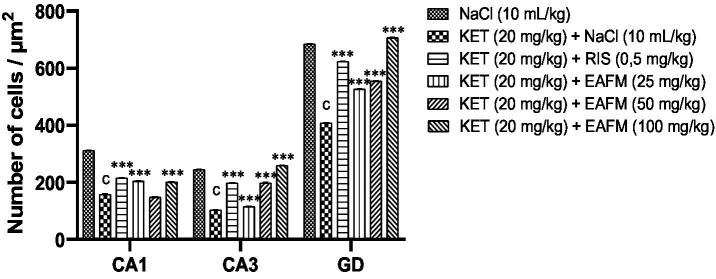
Effects of *Ficus mucuso* lyophilisate on the number of nucleated cells in the Ammon’s horn 1 & 3 and the dentate gyrus of the hippocampus. Each bar represents the mean ± SEM. *N* = 10; ^c^*p* < 0.001 compared to NaCl. ***p < 0.001 significant difference compared to KET. Two-way ANOVA followed by Bonferroni post hoc test. KET = Ketamine (20 mg/kg). NaCl = Saline solution (0.9%); RIS = Risperidone (0.5 mg/kg).

The cell count per μm^2^ of tissue in CA1 & CA3 and the dentate gyrus of the hippocampus is shown in Figure 13. Ketamine caused a significant decrease in the number of cells due to necrosis and multiplication of hyperchromatic cells.

Ketamine caused a significant decrease (*p* < 0.001) in the number of CA1 cells from 311.33 ± 1.14 of cells in the normal control to 157.83 ± 1.28 in the negative control. The aqueous lyophilisate of *F. mucuso* restored the neuronal structure by significantly increasing [*F*(5, 30) = 3542, *p* < 0.001] in the number of CA1 cells to 203.66 ± 1.42 and 200.16 ± 1.57 with doses of 25 and 100 mg/kg, respectively. Risperidone caused an increase (*p* < 0.001) in the number of cells to 214.83 ± 1.90 in CA1 compared to the negative control.

Ketamine caused a decrease (*p* < 0.001) in the number of nucleated cells in CA3, which fell from 244.5 ± 1.71 in the normal control to 102.66 ± 1.23 cells in the negative control. The aqueous lyophilisate of *F. mucuso* restored the neuronal structure through a significant [*F*(5, 30) = 4545, *p* < 0.001] dose-dependent increase in the number of CA3 cells to 114.66 ± 1.71; 198.33 ± 1.14 and 259.0 ± 1.71 with doses of 25, 50 and 100 mg/kg. Risperidone caused an increase (*p* < 0.001) in the number of CA3 cells to 197.33 ± 1.42.

Finally, ketamine caused a significant decrease (*p* < 0.001) in the number of nucleated cells in the dentate gyrus, which fell from 684.83 ± 1.85 in the normal control to 408.0 ± 1.71 in the negative control. The aqueous lyophilisate of *F. mucuso* (25, 50 and 100 mg/kg) significantly increased [*F*(5, 30) = 11824, *p* < 0.001] the number of cells in the dentate gyrus to 526.16 ± 1.61; 554.0 ± 1.71 and 706.33 ± 1.42, respectively. Risperidone caused an increase (*p* < 0.001) in the number of cells to 623.5 ± 1.57 in the dentate gyrus.

### 11.9 Effects of aqueous lyophilisate of *Ficus mucuso* on cells of the prefrontal cortex and striatum

**Figure 14:**
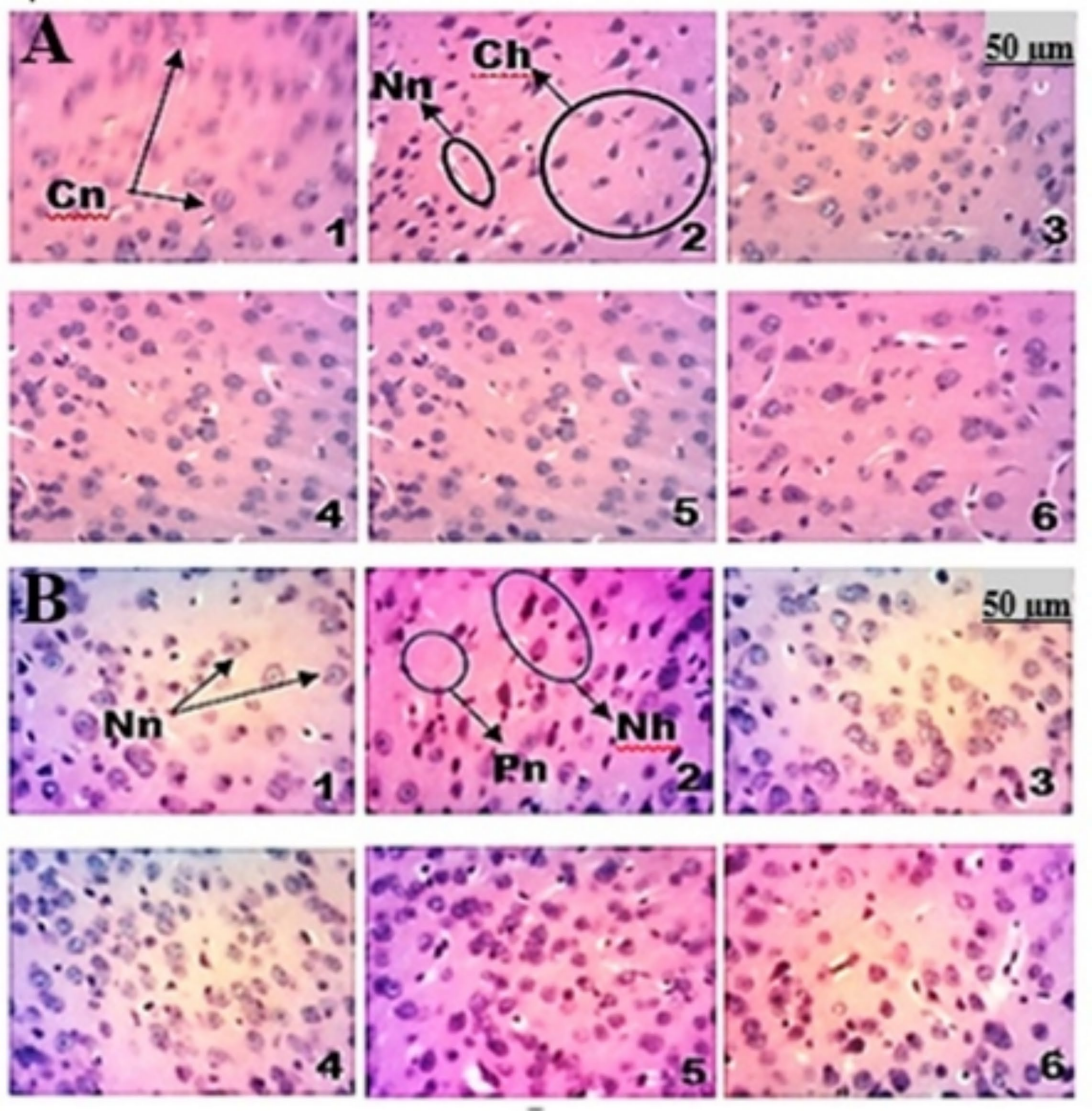
Microphotographs of the prefrontal cortex **(A)** and striatum **(B)**: (×250); Hematoxylin-eosin staining. 1 = Normal control (NaCl, 0.9%); 2 = Negative control (KET); 3 = Positive control (RIS); 4, 5, 6 = Groups receiving aqueous lyophilisate of *Ficus mucuso* (25, 50, and 100 mg/kg). NC = Normal cells; Nn = Necrotic neuron; HC = Hyperchromatic cells; NP = Neuronal loss.

Histological analysis of the prefrontal cortex and striatum in mice from the normal control group shows a normal microstructure (microphotograph 1) of the prefrontal cortex and striatum (Figure 14). In mice from the negative control group (microphotograph 2), histopathological changes were observed due to a loss of integrity of the neuronal tissue marked by hyperchromatic cells, necrosis and neuronal loss (Figures 14A,B). The groups receiving the aqueous lyophilisate at different doses of 25, 50 and 100 mg/kg (microphotographs 4, 5 and 6) and the group receiving risperidone (microphotograph 3) (Figures 14A,B) showed a microstructure of the prefrontal cortex and striatum with a healthy architecture and no lesions, with an absence of neurodegeneration similar to that of the normal control (microphotograph 1).

#### 11.9.1 Effects of aqueous lyophilisate of *Ficus mucuso* on the number of nucleated cells in the prefrontal cortex and striatum

**Figure 15:**
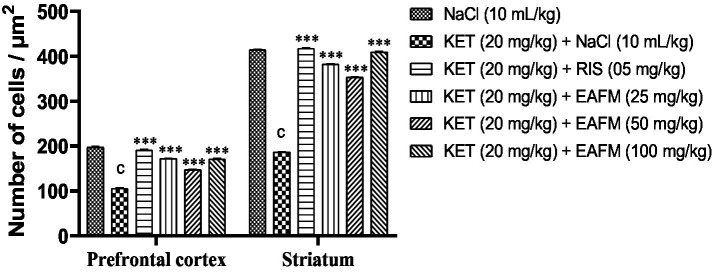
Effects of *Ficus mucuso* lyophilisate on the number of nucleated cells in the prefrontal cortex and striatum. Each bar represents the mean ± SEM. *N* = 10; *p* < 0.001 compared to NaCl. ***p < 0.001 significant difference compared to KET. Two-way ANOVA followed by Bonferroni post hoc test. KET = Ketamine (20 mg/kg). NaCl = Saline solution (0.9%); RIS = Risperidone (0.5 mg/kg).

Figure 15 shows that ketamine administration leads to the appearance of hyperchromatic cells and necrotic neurons in the prefrontal cortex and striatum (Figure 15). A significant decrease (*p* < 0.001) in cell number was observed, from 197 ± 2.57 in the normal control to 105.5 ± 2.28 in the negative control (KET). However, the aqueous lyophilisate of *F. mucuso* restored cell structure by significantly increasing [*F*(5, 30) = 447, *p* < 0.001] the number of cells in the prefrontal cortex to 172.83 ± 2.42; 147 ± 1.71 and 171 ± 2.57 at doses of 25, 50 and 100 mg/kg. Risperidone caused an increase (*p* < 0.001) in the number of cells to 190.66 ± 2.47.

A significant decrease (*p* < 0.001) in the number of ST cells was also observed, from 414.5 ± 1.28 in the normal control to 186.16 ± 1.90 in the negative control. However, the aqueous lyophilisate of *F. mucuso* significantly increased [*F*(5, 30) = 7,690, *p* < 0.001] the number of ST cells to 382.16 ± 1.61; 353.16 ± 1.33 and 409.33 ± 1.90 at all doses. Risperidone caused an increase (*p* < 0.001) in the number of ST cells to 417.33 ± 1.71 compared to the negative control (Figure 15).

## 12 Discussion

The aim of this study was to evaluate the antipsychotic effect of aqueous lyophilisate bark from the trunk of *F. mucuso* on ketamine-induced schizophrenia in albino mice. It appears that the central point of neurobehavioral change in schizophrenia is based on changes in the activities or release of neurotransmitters such as dopamine, serotonin, glutamate, acetylcholinesterase, adrenaline, aspartate, glycine, GABA and GABA-T (5, 10, 16, 18, 55, 56). The severity of schizophrenia depends in part on oxidative stress and the level of neurochemical imbalance affecting neural circuits in several brain structures such as the hippocampus, prefrontal cortex, and striatum (18, 57). The results of this study confirm those of previous research showing that repeated administration of ketamine causes positive symptoms, negative symptoms, and cognitive symptoms or disorders (55, 58). Ketamine is an antagonist of NMDA glutamate receptors. When ketamine binds to NMDA receptors, an imbalance occurs between the release of dopamine, glutamate, and GABA. Inhibition of NMDA receptors leads to stereotypical behaviors and locomotor hyperactivity. Blocking NMDA receptors located on GABAergic interneurons reduces their inhibitory activity; GABAergic interneurons are known to exert an inhibitory role on glutamatergic neurons through the release of GABA. The reduction in the activity of these GABAergic interneurons leads to excessive release of glutamate, which in turn stimulates the mesolimbic circuits to release more dopamine, causing delusions and hallucinations (8, 21). The literature has shown that in addition to causing a state of NMDA receptor hypofunction, ketamine also activates D_2_ receptors.

Stereotypy is used to diagnose the positive symptoms of ketamine-induced schizophrenia in mice (37, 59). These symptoms are a series of repetitive movements and gestures with no apparent purpose: head movements, intense licking, chewing, and intermittent sniffing (60). Previous studies have shown that the increase in the time and number of escalations is due to an increase in dopaminergic and glutamatergic activity in the cortical areas of the brain, which results in repetitive behaviors (1, 42). The aqueous lyophilisate of *F. mucuso* (25, 50, and 100 mg/kg) had a consistent calming effect on the time to climb and the number of climbs between 10 and 65 min. The reduction in the time and number of escalations reflects the simplification of the positive symptoms of schizophrenia. *F. mucuso* appears to have a stimulating effect on GABA receptors, which promotes a decrease in neuronal excitation by lowering dopamine levels (5). However, *F. mucuso* reduced ketamine-induced repetitive behavior between 10 and 65 min. Our results are consistent with those obtained for *Morinda citrifolia* (61), *Terminalia ivorensis* (42), *Scopoletin* and *Rutin* (62), and *Terminalia macroptera* (37), which reduced stereotypy in mice, demonstrating their antipsychotic effects.

Ketamine-induced hyperlocomotion is partly attributed to the blockade of NMDA receptors in the mesolimbic regions, leading to disinhibition and a consequent increase in neuronal excitation (63). In addition, ketamine acts as an indirect dopamine agonist, which explains the behavioral stimulation (64). The aqueous lyophilisate of *F. mucuso* (25, 50 and 100 mg/kg) attenuated the increase in locomotor activity by significantly reducing (*p* < 0.001) the number of lines crossed and the time spent moving. Consequently, *F. mucuso* is likely to be used in the treatment of the positive symptoms of schizophrenia. This is because it lifts the blockage of NMDA receptors on the inhibitory GABAergic system, often considered to be associated with the behavioral hyperactivity of schizophrenia (65). *F. mucuso* acts as GABA agonist, preventing the excessive release of dopamine in the hippocampus, prefrontal cortex, and striatum. Our results are consistent with those of (2, 37), who showed that extracts that reduce the number of lines crossed and increase immobility time are effective in reducing positive symptoms.

Negative symptoms of schizophrenia are a crucial feature of this disorder (66). Frequently present at the onset of the disease, they persist throughout its course, even during periods of remission. The increase in immobility induced by ketamine in the forced swim test in mice is a negative symptom (behavioral despair) characteristic of schizophrenia (2, 18, 67). Previous studies have reported the involvement of the 5-hydroxytryptaminergic (5-HT) system in the negative symptoms of schizophrenia (18, 68). The work of (69) indicates that increased immobility in forced swimming is caused by the blockade of 5-HT2A receptors. Similarly, the work of (38) showed that increased which showed that an increase in extracellular glutamate concentration causes hyperactivation of serotonin 5-HT2 receptors, responsible for the increase in immobility time induced by ketamine. The aqueous lyophilisate of *F. mucuso* (25, 50 and 100 mg/kg) significantly reduced (*p* < 0.001) immobility time in mice. The reduction in immobility in the forced swim test by the plant suggests the existence of antidepressant properties. Consequently, the lyophilisate acts as a 5-HT2A receptor antagonist and may have antipsychotic effects. Our results and observations are supported by previous studies conducted on *Terminalia ivorensis* (42), *Philenoptera cyanescens* (67), *Terminalia macroptera* (37), *Cissampelos owariensis* (59), and Diosgenin (2), which have shown that reduced immobility is a sign of reduced despair and negative symptoms in schizophrenia.

The Y-maze was developed to study learning and short-term memory functions in mice (41). Cognitive disorders, such as deficits in attention, language, executive functions, and working memory, are fundamental cognitive symptoms in schizophrenia (70). Ketamine-induced memory impairment is linked to antagonism of glutamatergic and acetylcholine neurotransmission and to stimulation of oxidative stress in the brain (2, 18). Increased acetylcholinesterase activity in the brain also confirms memory deficits in schizophrenia (21). Administration of *F. mucuso* lyophilisate (25, 50 and 100 mg/kg) reduced cognitive function impairments, as indicated by an increase in the number and percentage of correct alternations. Previous studies have reported improvements in cognitive deficits with antipsychotic drugs and plant extracts (7, 21, 37, 71). Mechanistically, learning is associated with phosphorylation by calcium/calmodulin-dependent protein kinase II (Ca^2+^/CaMK II). Inhibition of the NMDA receptor, which is a ligand-activated Ca^2+^ channel, decreases this phosphorylation, which could explain the cognitive deficits induced by ketamine due to the disruption of long-term potentiation (72). In the early stages of memory formation, ketamine also inhibits acetylcholine and antagonizes the α-7nAchR nicotinic acetylcholine receptor (73). Therefore, *F. mucuso* lyophilisate improves cognitive impairment through its ability to enhance working memory by stimulating the synthesis of acetylcholine and glutamate.

The hypofunctionality of dopaminergic neurotransmission in the prefrontal cortex is thought to be partly responsible for schizophrenia. This is due to the blockade of NMDA receptors in the ventral tegmental area, which leads to a reduction in cortical dopamine release. Ketamine is known to influence dopamine transmission and receptor activation via multiple mechanisms (74). It is important to note that ketamine has two major effects in the brain: it increases dopamine release in the striatum and inhibits its uptake in the same organ (42). In addition, one mechanism by which ketamine produces undesirable behavioral effects is related to the blockade of NMDA receptors located on GABAergic inhibitory neurons in the limbic and subcortical regions of the brain (75). This disinhibitory action would increase neuronal activity and cause excessive dopamine release in the limbic and striatal regions following dysfunction of the striatum, prefrontal cortex, and hippocampus, likely resulting in hyperdopaminergic and glutamatergic signaling in schizophrenia (76, 77). NMDA receptor antagonists are associated with increased dopamine and glutamate release, leading to reduced cortical inhibition. The accumulation of cytosolic dopamine can lead to neurotoxicity, a detrimental effect on neuron survival (78, 79). Ketamine induced an increase in dopamine levels in the brain (18, 80, 81). Administration of the aqueous lyophilisate (25, 50 and 100 mg/kg) normalized dopamine levels in the hippocampus, prefrontal cortex, and striatum by significantly reducing (*p* < 0.01; *p* < 0.001) dopamine levels. Our results corroborate previous studies (5, 21) which indicated that hypoglutamatergia is responsible for the decrease in cerebral dopamine. *F. mucuso* may have acted through the mechanism of dopamine regulation and therefore as an antagonist of D1 and D2 receptors.

Numerous studies have examined the neural circuits responsible for functional brain networks and cognitive processes (2). Several studies have shown that, in addition to alterations in dopaminergic and cholinergic transmission (82), the 5-HTergic system may also be a key factor primarily linked to negative symptoms in schizophrenia (21). Negative symptoms have been associated with an increase in serotonergic transmission dependent on 5-HT2A/2C receptors (18). Indeed, increased 5-HT2A receptor regulation is linked to exacerbated GABAergic phosphorylation, increased dopamine release, and glutamatergic excitotoxicity in the striatum (18, 83). Consequently, these disturbances could be the cause of behavioral abnormalities. Exposure to ketamine caused an abnormal increase 5-HT concentration in the hippocampus, prefrontal cortex, and striatum (4, 84). Previous studies have shown that the sustained loss of GABAergic interneurons induced by ketamine was linked to an upregulation of the enzyme responsible for the synthesis of superoxide radicals, which are responsible for stereotypical behaviors and depression (21, 85). However, administration of the aqueous lyophilisate of *F. mucuso* (25, 50 and 100 mg/kg) significantly decreased (*p* < 0.05; *p* < 0.01; *p* < 0.001) serotonin concentration in the striatal, prefrontal cortical, and hippocampal regions of the mouse brain. A decrease in 5-HT could be caused by an increase in the activity of the enzyme glutamate decarboxylase. The latter plays a role in regulating the synthesis of GABA from glutamate, suggesting an increase in GABAergic neurotransmission (2, 4). The improvement in negative deficits would be significantly influenced by the reduction in serotonin in the PFC, HPC and ST and therefore the plant would act as a 5-HT antagonist through its modulatory activity on the 5-HTergic system.

Alteration of glutamatergic neurotransmission pathways or inhibition of glutamate reuptake has been considered a determining factor in the severity of schizophrenic symptoms (86, 87). This hypothesis is supported by previous studies showing altered expression and function of glutamate transporters on astrocytes or calcium-dependent signaling in the pathophysiology of schizophrenia (87). This suggests that elevated levels of glutamate in certain brain regions or increased glutamate reuptake may contribute significantly to schizophrenia (5, 55). Impaired calcium-dependent glutamate release by glutamatergic neurons has been associated with disturbances in plasma membrane calcium ATPase (5). In addition, ketamine induces a state of hypofunction of the NMDA receptor and its downstream signaling pathways, leading to disruption of glutamate activity toward other receptors, which is linked to mitochondrial dysfunction causing hyperstimulation (15, 88). Ketamine can therefore cause excessive calcium influx into the cytosol and glutamate-mediated excitotoxicity (87). A significant flow of glutamate has been observed in the prefrontal cortex and a decrease in the hippocampus and striatum, leading to dysfunction of the cortical and striatal pathways (10, 87, 89). Administration of aqueous lyophilisate of *F. mucuso* (25, 50 and 100 mg/kg) led to a decrease in glutamate levels in the hippocampus and striatum, and the opposite effect in the prefrontal cortex. Our results corroborate those of (5). This shows that the plant has antipsychotic properties.

Research has established that the positive, negative, and cognitive symptoms of schizophrenia have one thing in common: neurotransmitter imbalance and disruption of connectivity between the hippocampus, striatum, and cortex (90). Abnormalities in cholinergic transmission have been identified as being associated with the cognitive impairment observed in schizophrenia (2, 9). These involve increased acetylcholinesterase concentration in the prefrontal cortex, hippocampus, and striatum (21, 89, 91). This abnormality has been observed during chronic administration of ketamine (56). Acetylcholinesterase hyperactivity causes cognitive symptoms such as memory and learning disorders (92). This further confirms ketamine ability to influence α7nAChR receptors and other regulatory molecules essential for learning and memory (72). Ketamine causes an increase in cholinesterase levels in the hippocampus, which leads to the suppression of acetylcholine and glutamate and causes cognitive symptoms in mice. The imbalance in cholinergic neurotransmission related to cognitive function is the result of dysregulated acetylcholinesterase activity (84). Ketamine treatment increased AchE flow in the HPC, PFC and ST and reduced Ach concentration, thereby attenuating cholinergic transmission and suppressing nicotinic and muscarinic receptor activities. The aqueous lyophilisate of *F. mucuso* (25, 50, and 100 mg/kg) reversed this effect by significantly reducing (*p* < 0.001) AchE activity, clearly suggesting an increase in the hippocampal, cortical, and striatal cholinergic system. Furthermore, low AchE levels are linked to cholinergic improvement. The ability of *F. mucuso* to reduce AchE activity, which is responsible for the degradation of acetylcholine, suggests increased cholinergic neurotransmission in the prefrontal cortex and hippocampus. Our results are consistent with the work of (2, 5, 91), which showed that the improvement in memory functions is due to a reduction in AchE levels in the brain.

Ketamine-induced NMDA receptor blockade causes GABAergic inhibition, leading to a decrease in gamma-aminobutyric acid (GABA) levels and an increase in neuronal activity, resulting in excess dopamine and serotonin in schizophrenia (91). The administration of ketamine to mice resulted in a reduction in GABA release and, therefore, an increase in GABA-T activity and dopamine release, which stimulates stereotypy and hyperlocomotion, which are positive symptoms (2, 93). This enzyme decreases the level of GABA in the brain and at the same time increases the level of the excitatory neurotransmitter glutamate, causing hyper-excitation of neurons (94). Increased GABA levels and decreased GABA-T activity after administration of different doses (25, 50 and 100 mg/kg) of the aqueous lyophilisate of *F. mucuso* were observed in different regions of the brain. These findings suggest a close interaction between the lyophilisate and GABAergic neurotransmission. These results show that the lyophilisate may be capable of restoring and maintaining the balance between the inhibitory system by downregulating dopamine in the prefrontal cortex. The plant acts as a GABA receptor agonist and GABA-T antagonist, inhibiting voltage-dependent sodium channels and increasing GABA concentration in the synapses, thereby facilitating GABAergic neurotransmission. *F. mucuso* appears to act as a tranquilizer. Our results are consistent with those obtained for *Lippia multiflora* (95) and *Cissus quadrangularis* (90).

Under normal conditions, the human body has an oxidative system and an antioxidant defense system, both of which are in dynamic equilibrium. In abnormal situations, free radicals that are not eliminated from the body can react with normal tissue cells, damaging cell structure and causing cell dysfunction. Oxidative stress occurs when the body is subjected to various internal and external environmental stimuli, leading to an imbalance between oxidative and antioxidant processes (6). Moderate oxidative stress is necessary for normal physiological functioning of the body, while excessive oxidative stress can have deleterious effects. The main targets of oxidative stress damage are DNA, lipids and proteins (96). Ketamine administration induced a state of NMDA receptor hypofunction, causing glutamate activity to shift to other receptors, resulting in hyperstimulation (89). It causes excessive cytosolic Ca^2+^ influx and glutamate-mediated excitotoxicity (87). The brain accounts for 2% of total body weight but consumes about 20% of the body oxygen. Compared to other organs, it consumes more oxygen, generates more free radicals, and has lower levels of antioxidants, making it more susceptible to damage from oxidative stress (96). Social isolation, vitamin D deficiency, chronic stress during adolescence, perinatal infections, inflammation, etc. all ultimately lead to the oxidative stress observed in schizophrenia (97). Various studies have established a link between impaired glutamate metabolism and the oxidative stress observed in the brains of schizophrenics. Glutamate is converted to GSH via a two-step enzymatic process requiring ATP (glutamate-cysteine ligase and GSH synthase) and metabolized by the enzyme glutamate decarboxylase (GAD) to GABA, the main inhibitory neurotransmitter (21). Thus, alterations in the GAD biosynthesis pathway and GSH synthesis are associated with redox imbalance, as evidenced by elevated MDA concentrations (7, 86). This corroborates several previous studies showing that repeated administration of ketamine in mice increased lipid peroxidation (MDA) and reduced SOD, GSH and CAT activity (2, 5, 98, 99). Similarly, ketamine causes an increase in nitrite levels, associated with nitrergic stress. The increase in nitrite concentration in the brain was associated with an increase in dopamine and glutamate release through upregulation of calcium-induced nitric oxide synthesis mediated by NMDA receptors (5, 91). However, treatment with the aqueous lyophilisate of *F. mucuso* reversed these changes in the hippocampal, striatal, and cortical areas by increasing GSH levels and SOD and CAT activities. Nevertheless, *F. mucuso* reduced markers of lipid peroxidation (MDA) and nitrergic stress (NO) in the HPC, PFC and ST. Risperidone also increased antioxidant levels and decreased pro-oxidant levels (2, 91). Oxidative stress is recognized as a pathological pathway involved in the alteration of NMDA receptor response due to changes in GSH biosynthesis (100). It should be noted that the increase in GSH concentration may reflect a modulatory action of neurotransmitters, due to the components of GSH (glutamate, glycine, and cysteine). Our results corroborate those of (2, 5, 80), which showed that reduced levels of MDA and NO and increased levels of CAT, SOD and GSH in the HPC, PFC and ST reflect a reduction in ketamine-induced oxidative stress. The plant acts through the oxidative pathway, protecting the brain against excitotoxicity and alteration of neuronal structure.

Brain alterations were observed in microphotographs of the HPC, PFC and ST, confirmed by neuronal loss due to leukocyte infiltration, cytolysis, and necrosis in schizophrenic mice. Necrosis, hyperchromatic cells and cell degeneration were observed in CA1 and CA3. Pyknosed cells, degeneration of the pyramidal and radiatum layers, and cell death due to the action of endocellular enzymes were observed in CA1. Necrosis of the polymorphic, granular, and molecular layers was observed in the dentate gyrus. All of the above explains the neurodegeneration and neuronal death due to oxidative stress and neurochemical alteration induced by ketamine. *F. mucuso* (25, 50 and 100 mg/kg) restored neuronal integrity in CA1, CA3, and GD in the HPC, ST and PFC.

## 13 Conclusion

This study evaluated the antipsychotic effects of the aqueous lyophilisate of *F. mucuso* bark on behavioral disturbances, oxidative stress, neurochemical deficits, and neurodegeneration induced by ketamine in mice. The results as a whole demonstrate the role of the aqueous lyophilisate of *F. mucuso* in managing stereotypy, hyperlocomotion, despair, and memory loss in schizophrenia. The reversal of behavioral disorders in schizophrenia by *F. mucuso* is mediated by the modulation of neurochemical transmission and the mechanism of oxidative and nitrergic stress inhibition. The plant repaired brain damage by restoring the integrity of neuronal cells in the hippocampus, prefrontal cortex, and striatum in mice. In addition, it stimulated the production of new cells. All these observations confirm the use of *F. mucuso* as an antipsychotic for the management of schizophrenia.

## Data Availability

The raw data supporting the conclusions of this article will be made available by the authors without undue reservation.
